# Polymer Conformations, Entanglements and Dynamics in Ionic Nanocomposites: A Molecular Dynamics Study

**DOI:** 10.3390/polym12112591

**Published:** 2020-11-04

**Authors:** Ahmad Moghimikheirabadi, Clément Mugemana, Martin Kröger, Argyrios V. Karatrantos

**Affiliations:** 1 Polymer Physics, Department of Materials, ETH Zurich, Leopold-Ruzicka-Weg 4, CH-8093 Zurich, Switzerland; 2 Materials Research and Technology, Luxembourg Institute of Science and Technology, 5, Avenue des Hauts-Fourneaux, L-4362 Esch-sur-Alzette, Luxembourg; clement.mugemana@list.lu

**Keywords:** ionic interactions, entangled polymers, charged nanoparticles, structure, crosslinks, dynamics, entanglements

## Abstract

We investigate nanoparticle (NP) dispersion, polymer conformations, entanglements and dynamics in ionic nanocomposites. To this end, we study nanocomposite systems with various spherical NP loadings, three different molecular weights, two different Bjerrum lengths, and two types of charge-sequenced polymers by means of molecular dynamics simulations. NP dispersion can be achieved in either oligomeric or entangled polymeric matrices due to the presence of electrostatic interactions. We show that the overall conformations of ionic oligomer chains, as characterized by their radii of gyration, are affected by the presence and the amount of charged NPs, while the dimensions of charged entangled polymers remain unperturbed. Both the dynamical behavior of polymers and NPs, and the lifetime and amount of temporary crosslinks, are found to depend on the ratio between the Bjerrum length and characteristic distance between charged monomers. Polymer–polymer entanglements start to decrease beyond a certain NP loading. The dynamics of ionic NPs and polymers is very different compared with their non-ionic counterparts. Specifically, ionic NP dynamics is getting enhanced in entangled matrices and also accelerates with the increase of NP loading.

## 1. Introduction

Polymer nanocomposites have received special attention from academia and industry during the last 30 years, due to their improved properties [1,2,3,4,5,6,7] in comparison to polymer blends. Nanoparticle (NP) dispersion [8,9,10,11,12,13,14,15] is necessary for effective reinforcement [9,16] in the matrix and is a prerequisite for property “tuning” [17] and enhancement. There is strong motivation to explore and interrelate polymer conformation, entanglements, dynamics and structure, since these can influence macroscopic properties. One way to provide NP dispersion is to let the interaction between NPs and chains to be of an ionic nature [18,19,20]. The presence of oppositely charged ions at the polymer/nanofiller interphase can promote dispersion [21,22,23,24,25]. This relatively new class of ionic nanocomposites combines filler reinforcement with the reversibility of ionic interactions [23]. Despite the improved mechanical performance, the reversible feature of ionic bonds, that can break and reform under certain conditions [21], has led to smart materials for self-healing [25,26], shape-memory [21,22], piezoelectric [27], and mechanochromic [28] applications due to their ability to respond directly under a certain chemical or physical stimulus. For instance, nanocomposites based on imidazolium-functionalized polyurethane and surface-modified sulfonate silica (SiO${}_{2}$) NPs have revealed not only unprecedented mechanical properties with simultaneous improvements in stiffness, toughness and extensibility but also a scratch recovery ability [21]. A blend of surface-ionic charged silica and imidazolium chain-ends functionalized polylactide (PLA) and poly[ϵ-caprolactone-co-d,l-lactide] (P[CL-co-LA]), has led to ionic nanocomposites with a shape-memory property, due to the dynamic nature of the ionic interactions [21,22,29,30]. Commercially available PLA were blended with polyurethane-grafted imidazolium moieties and surface-modified sulfonated silica NPs resulting in ultra-tough PLA ionic nanocomposites [23]. Such composites offer a broad range of application, including antibacterial surfaces that can prevent bacterial attachment and biofilm formation as a result of the ionic charges within the interphase between the polymer matrix and the NP surface [31].

NPs functionalized with ionically modified coronas in matrix-free “nanocomposites”, such as hairy NP assemblies, are able to homogeneously disperse [19,32,33]. In addition, highly dispersed ionic silica and poly(ethylene oxide) nanocomposites were made by mixing end-functionalized amine-terminated polymers (PEO-NH${}_{2}$) with the inherently acidic hydroxyl groups present on the surface of silica NPs [18]. Adding nanosilica to the PEO-NH${}_{2}$ led to negatively charged silica from the reaction between the protons from the silanols with the terminal amine groups on the PEO. As a result, an exceptional degree of dispersion of the silica in the polymer and high degree of order in both thin film and bulk forms was achieved [18]. For matrix-free model systems [34,35] it was observed that NPs diffuse similarly to a polymer solution [36,37,38], while chains diffuse faster than NPs [39]. Ionic interactions are also included in the case of ionomers in which the morphology and phase behavior depend on the electrostatic strength [40,41,42]. In particular, ionic clusters are formed by increasing the electrostatic strength developed from linear to branched structures [40]. In a simulation of ionomer (poly(ethylene-co-methacrylic acid)) nanocomposites [43], it was observed that ions were depleted around a neutral NP, but they were denser relative to the bulk value in the case of a sticky NP. In addition, ionomers move closer to the NP under conditions of high ion density [43].

Despite their potential applications, and to the best of our knowledge, no fundamental research has been performed to date on ionic entangled polymers reinforced with ionic NPs in order to understand and investigate the ionic interactions, interphasial region, and NP dispersion state of the nanocomposite. In particular, we are not aware of any experimental measurements on polymer dimensions, structure or dynamics in ionic nanocomposites. We set out to investigate how the ionic (electrostatic) interaction between NPs and entangled polymers impact the NP dispersion state, polymer structure, dimensions, dynamics, and entanglements.

This paper is organized as follows: In Section 2, the methodology and simulation details of the present study are described. We begin by investigating the structure of polymers and NPs in the polymer melt (Section 3.1). We report the polymer dimensions of all the nanocomposites studied as a function of NP loading and different polymer charge density in Section 3.2. Subsequently, in Section 3.3 we calculate and compare the dynamics of NPs for all nanocomposites, where good NP dispersion can be achieved for different polymer charge densities. We further study chain–NP crosslinks, and their lifetime distributions (Section 3.4). Section 3.5 is devoted to the analysis and role of entanglements present in the nanocomposites. Finally, in Section 4, conclusions are presented.

## 2. Methodology

Full atomistic simulation of a polymer ionic nanocomposite is out of reach nowadays. We are therefore using a coarse-grained model that is known to capture the relevant dynamics and structure of simple polymeric systems and detailed enough to study the effect of charge sequence on material behavior. There are a few attempts that aim at coarse-graining similar but uncharged systems based on atomistic simulation [16,44,45,46].

**Figure 1:**
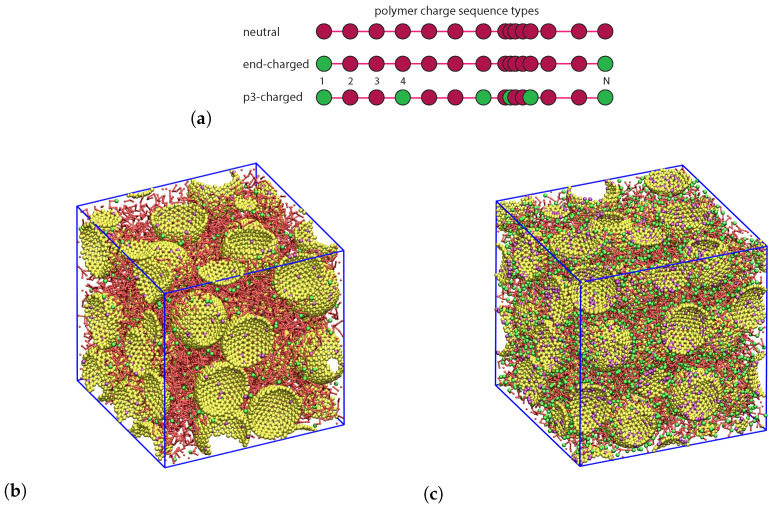
(**a**) The three polymer charge sequence types considered in this work. Each green bead carries a single positive charge *q*. (**b**,**c**) Equilibrium 3D orthographic snapshots at a nanoparticle (NP) volume fraction of *ϕ* ≈ 37%. (**b**) Non-dispersed system of end-charged *N* = 40 chains, and (**c**) dispersed system of periodic p3-charged *N* = 100 chains. The former system (**b**) consists of 27 NPs and 270 chains in a simulation box with dimensions of 27.6 × 27.6 × 27.6, and the latter (**c**) of 36 NPs, 144 chains in a simulation box of the size 30.34 × 30.34 × 30.34. Green spheres show positively charged monomers, and purple spheres the negatively charged NP surface beads. Neutral NP surface beads and monomers are shown by yellow and (small) red spheres respectively. Bond connections between successive monomers within the chains are shown by red cylinders.

Our model systems (Figure 1) are composed of spherical NPs and multibead-spring linear polymer chains (so-called Kremer–Grest model [47]), where each bead represents a number of monomers [48,49]. Adjacent beads within chains are connected by anharmonic springs, while the impenetrable NPs are modeled as rigid, mobile objects of which the surfaces are covered by beads. Polymeric and surface beads may or may not carry a permanent charge, while the systems are overall neutral in each case. We use Lennard–Jones (LJ) units throughout this manuscript, so that all quantities are given as non-dimensional numbers. They receive their dimensional counterparts through their physical units and the dimensional length, time, mass, and charge units, as described in Table 1, which we (can) assign afterward [50], so that all results presented hold up for arbitrary choices of real materials.

To be more specific, adjacent beads *i* and *j* separated by spatial distance $r_{ij}$ within polymer chains are connected using finitely extendable nonlinear elastic (FENE) springs [47,51,52,53,54,55,56]:

(1) $$V_{ij}^{\mathrm{FENE}}=-\frac{1}{2}kR_{0}^{2}\ln\left(1-\frac{r_{ij}^{2}}{R_{0}^{2}}\right),$$

when in applying Equation (1), the maximum bond length and spring coefficient were set to $R_{0}=1.5$ and k=30, respectively, as in previous works on neutral polymers [47,52]. All beads interact via a truncated, purely repulsive LJ potential $V_{ij}^{\mathrm{LJ}}$, the corresponding force of which acts along the line between the centers of mass of two particles [57]. It is given by:

(2) $$V_{ij}^{\mathrm{LJ}}=4\left(\frac{\sigma_{ij}^{12}}{{r_{ij}}^{12}}-\frac{\sigma_{ij}^{6}}{{r_{ij}}^{6}}\right),\;r_{ij}\leq 2^{1/6}\sigma_{ij},$$

where $r_{ij}$ represents the spatial distance between any beads i≠j. The entanglement length [58] of this model is $N_{e}\approx 86$ by the modified S-coil estimator [59], the bond length is approximately unity, and the characteristic ratio is $C_{\infty }\approx 1.7$ at our chosen temperature T=1.25 (Supplementary Figure S1b). The Lorentz–Berthelot mixing rule $\sigma_{ij}=\left(\sigma_{i}+\sigma_{j}\right)/2$ [57] is used; $\sigma_{i}=1$ if particle *i* belongs to the set of monomers, and $\sigma_{i}=0.4$ if *i* belongs to the set of surface beads of the NPs. In addition, the coulombic interaction between charged beads in the polymer matrix and on the NP surface is incorporated and given by:

(3) $$V_{ij}^{\mathrm{Coulomb}}=\frac{q_{i}q_{j}}{\varepsilon_{r}r_{ij}}$$

with $q_{i}=+q$ for charged monomers and $q_{i}=-q$ for charged NP surface beads, where *q* is the elementary charge of proton *e* in LJ units, i.e., $q=e/\sqrt{4\pi \varepsilon_{0}\sigma \epsilon }$. Here, $\varepsilon_{r}$ denotes the effective relative dielectric constant of the composite system, which is usually deduced from the dielectric constants of the two constituents by a mixing expression such as the Maxwell–Garnett mixing rule based on an effective-medium theory [60,61]. Many non-conjugated polymers have a low relative dielectric constant, which decreases with the molecular weight and temperature, near nanosilica $\varepsilon_{r}$ value, thus we adopt the same $\varepsilon_{r}$ value, in this study, for polymers and NP fillers as in previous studies [34,35], and do not consider any ϕ-dependence of the effective $\varepsilon_{r}$. Our choice for $\varepsilon_{r}$ will be discussed further below. All NPs are equally and negatively charged; *Q* of its surface beads carry a negative charge −q. The molecular dynamics simulations were performed using the LAMMPS package [62]. The long-range electrostatics were computed using the particle–particle particle–mesh (PPPM) method [41] with an accuracy of $10^{-4}$. Details of the ionic nanocomposite systems studied, including NP volume fraction ϕ (%), the number of spherical NPs $n_{\mathrm{NP}}$, polymerization degree *N*, number of *n* chains, individual NP negative charge *Q*, and charge distribution on chains (Figure 1a), are summarized in Table 2.

The modeled polymer nanocomposites consist of spherical rigid NPs with the baseline radius of 3.75 (implying an effective NP radius of $r_{\mathrm{NP}}=3.75+0.7/2=4.1$), and fully covered with 720 surface beads. The NPs used in our study are hollow. Such an NP model is used to mimic nanosilicas in a dense melt, and has been utilized in other studies [63,64] as well. In experiments of ionic nanocomposites, nanosilicas are surface modified with a sulfonate group, SO${}_{3}^{-}$, that carries the charge [21,22]. We thus distribute the charge of an NP over its surface. A snapshot of a modeled nanocomposite, created by means of VMD software [65], can be seen in Figure 1. The mass of the NP surface beads, $m_{\mathrm{NP}}=0.49$, is chosen so that the NP mass density, calculated as $\rho_{\mathrm{NP}}=720\times m_{\mathrm{NP}}/V_{\mathrm{NP}}$ with $V_{\mathrm{NP}}=4/3\pi r_{\mathrm{NP}}^{3}$, is ≈1.5 times the mass density of the polymer matrix with monomer mass of m=1, calculated from ρ=nNm/V(1−ϕ) with the simulation box volume *V*, and NP volume fraction $\phi =n_{\mathrm{NP}}V_{\mathrm{NP}}/V$. All simulations were started from relaxed configurations of conventional non-ionic nanocomposites [66] with all Coulomb interactions turned off, at pressure P=4.84 and temperature T=1.25, resulting in equilibrium mass densities of ≈0.82 for monomers and ≈1.22 for NPs. The linear size of the simulation cell was always larger than the root mean square end-to-end distance of the polymer chains. We performed a system size analysis and found that the nanocomposite systems considered throughout this study are indeed large enough, thus the finite-size effects are negligible (see Supplementary Figures S7 and S8). Subsequently, the charges were assigned to the monomers and NP surface beads, and NVT simulations were performed at T=1.25 by means of a Nosé–Hoover thermostat with a damping time of 0.4 [56,67]. A time step equal to Δt=0.004 was used for polymer melts and nanocomposites. For some cases with relatively large charge density on the NP surface, a smaller timestep of Δt=0.002 was used.

The Bjerrum length $\lambda_{B}$ indicates the length scale at which the magnitude of the electrostatic interaction equals the thermal energy. It is thus obtained from Equation (3) as

(4) $$\lambda_{B}=\frac{q^{2}}{\varepsilon_{r}T}.$$

In the following, we estimate and then fix the Bjerrum length $\lambda_{B}$ through an (approximate) mapping of the simulated systems with existing materials. Imidazolium-functionalized polyurethane (PU)/silica [21] and poly(ethylene oxide) (PEO)/silica [68] nanocomposites have been studied in the melt state at around T≈333 K, thus implying $\epsilon \approx 266k_{B}$, and the nanosilica diameter of around 10 nm that results in σ≈1 nm. Therefore, the parameter *q*, which is the ratio between the elementary charge *e* and LJ charge unit as described in Table 1, is calculated and set as q≈7.92. While depending on molecular weight and the temperature the dielectric constant of PEO melt varies, we consider an effective dielectric constant of $\varepsilon_{r}=24$, which is around the values reported in [69,70], implying a Bjerrum length of $\lambda_{B}\approx 2.1$. For our p3-charged system this choice is compatible with a Manning number [71] (ratio between Bjerrum length and mean spatial distance between charges) close to unity, if the above values for $C_{\infty }$ and bond length are used. We also study the effect of strength of electrostatic interactions by simulating a set of replicate systems with a different effective dielectric constant of $\varepsilon_{r}=12$, or equivalently a stronger Coulomb interaction with a Bjerrum length of $\lambda_{B}\approx 4.2$.

## 3. Results and Discussion

### 3.1. Nanoparticle and Polymer Structure

**Figure 2:**
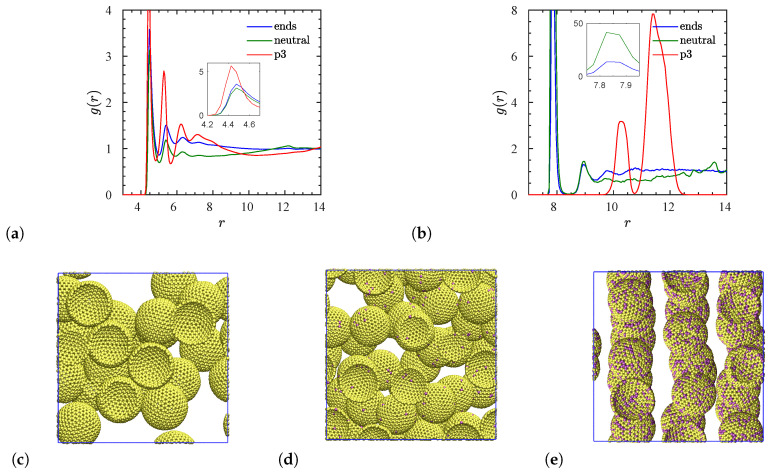
Radial distribution functions *g*(*r*) versus *r* in reduced LJ units (length unit *σ* ≈ 1 nm) for a system at NP volume fraction *ϕ* = 28.2% containing chains with a degree of polymerization *N* = 100. (**a**) NP center–monomer and (**b**) NP center–NP center distribution functions for the three types of chains. The insets show the corresponding values in the vicinity of the first peak in *g*(*r*). Only with p3-charged chains the NP dispersion is achieved for this choice of *N* and *ϕ*. The bottom row shows corresponding top-view snapshots of systems for (**c**) neutral, (**d**) end-charged, and (**e**) p3-charged chains. Only NPs are shown, and polymers are hidden, for the sake of clarity. Yellow and purple spheres represent neutral and charged surface beads, respectively.

Having equilibrated all the ionic nanocomposites we are in the position to analyze their structural and morphological properties based on the available trajectories of monomers and NPs. In particular, we show in Figure 2 the radial distribution functions (RDFs) of the NP center–monomer and NP center–NP center for the three different types of polymers (neutral, end-charged, and p3-charged) all with N=100, at an NP volume fraction of ϕ=20.8%. As can be seen, the NP center–monomer RDFs exhibit a surface layering structure for all types of chains. For neutral polymers, the first peak of the NP center–NP center (Figure 2b), at distance r=8, is much higher than the first peak of NP center–monomer (Figure 2a), signaling strong aggregation as indicated in Figure 2c. We observed such aggregation in all neutral (non-ionic) nanocomposites simulated in this study. In experiments of conventional (non-ionic) nanocomposites (such as polystyrene/silica nanocomposites), a phase separation has been observed when $R_{g}\leq r_{\mathrm{NP}}$ [72,73]. In addition, in polyurethane/silica nanocomposites, phase separation is observed when there is no charge functionalization of the PUs and nanosilicas [21]. In our case, for polymers carrying charges on their terminals, the NP center–NP center first peak of the RDF (Figure 2b) has a value large compared with the first NP center–monomer peak of the RDF (Figure 2a). This means that NPs are clustered (not dispersed) (Figure 2d) in the nanocomposite. The extent of clustering depends on the matrix molecular weight as was also observed by a previous study [74]. To be specific, clustering is observed for NP volume fractions ϕ>11.6% for matrices composed of end-charged N=100, 200 polymers. However, NP dispersion is observed in the presence of an unentangled matrix (end-charged N=40) up to ϕ=28.2%. In stark contrast, for p3-charged polymers, there is no NP center–NP center peak at *r* corresponding to the NP diameter (r=8), suggesting a nice dispersion of the NPs in these polymer matrices. In this case, there are no contacts between NPs. Under such conditions, NPs prefer to be in contact with the polymers. For such nanocomposites, a very strong layering can be observed for both RDFs, denoting a well defined interphase around NPs (Figure 2a) and a snapshot of them is depicted in Figure 2e. We observe a very similar interfacial behavior for the remaining polymer molecular weights (results not shown).

**Figure 3:**
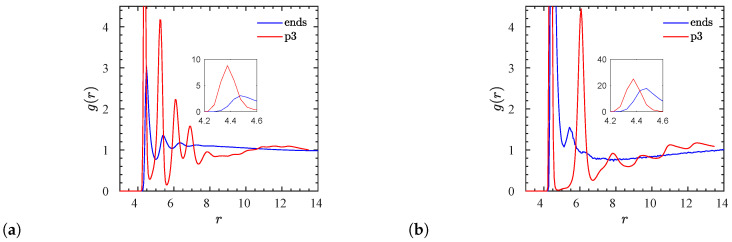
(**a**) NP center–monomer and (**b**) NP center–charged monomer radial distribution functions for dispersed systems with a degree of polymerization *N* = 200, and an NP volume fraction of *ϕ* = 11.6%. The insets show the corresponding values of the first peak in *g*(*r*).

Next, we focus on the radial distribution functions (RDFs) of the NP center–monomer and NP center–charged monomer for the two different types of charged polymers with N=200, at an NP volume fraction of ϕ=11.6%, where NPs in both systems are dispersed. We can clearly observe from Figure 3 that the NP center–charged monomer RDF presents a much higher value of the first peak (inset of Figure 3b) than that in the NP center–monomer RDF (inset of Figure 3a) [74]. This shows that there are more monomer contacts between the charged monomers and oppositely charged surface beads, than neutral monomers and neutral surface beads, due to the electrostatic attraction. In addition, the RDF amplitude of the second layer of the monomers around the NP center exhibits almost the same value for either charged or neutral monomers, whereas only the neutral monomers form more layers (3rd, 4th layers) around the NP center. For end-charged polymers, the monomer layering is much less significant farther away from the NP center.

**Figure 4:**
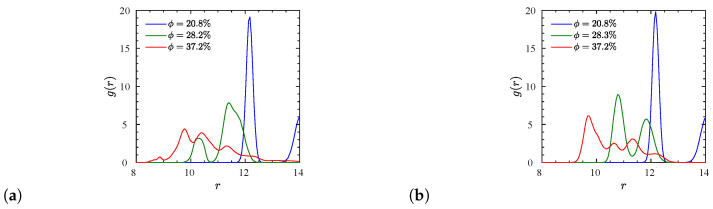

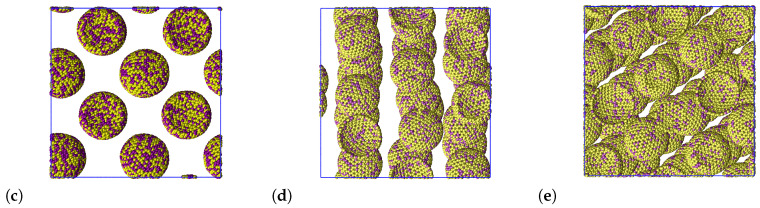
NP center–NP center radial distribution functions for dispersed p3-charged systems with a degree of polymerization (**a**) N=100 and (**b**) N=200. The bottom row shows the corresponding top-view snapshots of N=100 systems at the NP volume fractions of (**c**) ϕ=20.8%, (**d**) ϕ=28.2%, and (**e**) ϕ=37.2%. Only the NPs are shown here for the sake of clarity. Yellow and purple spheres stand for the neutral and charged surface beads respectively. 3D views of the systems shown in (**c**–**e**) are available in Supplementary Figure S9.

The dispersion of the matrices with p3-charged polymers is evident from the NP center–NP center RDFs for different NP volume fractions in Figure 4. Specifically, for the case of ϕ=20.8% there is a pronounced crystal-like structure of NPs in the matrix, as can already be deduced from the high amplitude of the first (blue) peak in the RDF (Figure 4a,c). This structural ‘perfection’ moderately degrades when the NP loading is increased, as observed by the decrease of the amplitude of the largest RDF peak (Figure 4a,e). Because the neighborhood remains intact, it comes at the expense of the occurrence of additional side peaks. While it is not obvious from the projections shown, our 3D visual analysis reveals that the structures are similar to a cubic body centered lattice at ϕ=20.8% (Figure 4c), hexagonal closest packing at ϕ=28.2% (Figure 4d), and a cubic face centered lattice at the largest ϕ=37.2% (Figure 4e). Supporting snapshots are available in Supplementary Figure S9.

### 3.2. Polymer Dimensions

**Figure 5:**
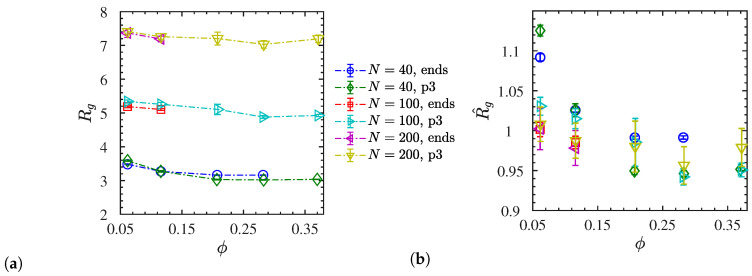
(**a**) Radii of gyration *R*_*g*_ for dispersed systems, (**b**) their normalized values ${\hat{R}}_{g}=R_{g}/R_{g}^{\mathrm{neutral}}$, both versus NP volume fraction. Dash-dotted lines are guides to the eye.

Here we focus our attention on the analysis of the size of polymers [72] residing in ionic nanocomposites. The analysis will be performed exclusively on nanocomposites in which the NPs are able to disperse in the polymer matrix. The squared radius of gyration $R_{g}^{2}$ of a molecule, defined as the average squared distance between monomers and their molecule’s center of mass in a given conformation is given by [49,66]:

(5) $$R_{g}^{2}\left(N\right)=\frac{1}{N}\sum_{i=1}^{N}\langle {\left(r_{i}-r_{\mathrm{cm}}\right)}^{2}\rangle ,$$

where $r_{\mathrm{cm}}=N^{-1}\sum_{i=1}r_{i}$ is the instantaneous center of mass of a chain. In Figure 5a, we show the $R_{g}$ of the polymers for both unentangled and entangled chains for both types of charged polymers as a function of the NP volume fraction when NP dispersion is achieved. The corresponding normalized values ${\hat{R}}_{g}=R_{g}/R_{g}^{\mathrm{neutral}}$ are shown in Figure 5b. We note here that for neutral chains, we do not observe any variation in $R_{g}$ (within statistical uncertainty) at different NP loading and polymer molecular weight. The systems behave like ideal chains with ${\langle R_{g}\rangle }^{2}\approx 0.265N$, see Supplementary Figure S1. We can observe in Figure 5b, that in entangled polymers (N=200), $R_{g}$ remains unperturbed by NP loading up to ϕ≈37% when $R_{g}\leq 2r_{\mathrm{NP}}$. However, for unentangled polymers (N=40), $R_{g}$ perturbs by ≈10% at ϕ≈6% and very slightly at ϕ≈11%. For higher NP volume fractions $R_{g}$ (ϕ>15%), we do observe polymer contraction for unentangled polymers (N=40), as can be seen clearly in Figure 5b. Although there are no $R_{g}$ experimental measurements for ionic nanocomposites, there are a few small angle neutron scattering (SANS) and small x ray scattering (SAXS) experimental measurements in conventional (non-ionic) polymer/silica [72,75,76,77] or polyhedral oligomeric silsesquioxane (POSS) [78] nanocomposites. In particular for polymer/silica nanocomposites, it was concluded that the $R_{g}$ was not altered by nanosilicas with up to 32% nanosilica loading if $R_{g}\leq 4r_{\mathrm{NP}}$ [72]. Only in the poly(ethylene-alt-propylene) (PEP)/silica nanocomposites has $R_{g}$ of PEP chains been observed to contract at very high nanosilica loading (ϕ≈50%) when $R_{g}>r_{\mathrm{NP}}$ but it remained unperturbed when $R_{g}\leq r_{\mathrm{NP}}$.

### 3.3. Chain Relaxation and Nanoparticle Mobility

**Figure 6:**
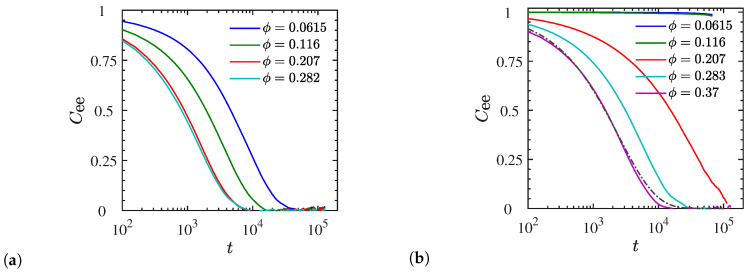
Chain end-to-end vector autocorrelation function $C_{\mathrm{ee}}\left(t\right)$ of (**a**) end-charged and (**b**) p3-charged *N* = 40 chains for dispersed systems at various NP volume fractions. Dash-dotted line in (**b**) shows the stretched-exponential fit to the numerical data of *ϕ* = 37%. In every case $C_{\mathrm{ee}}\left(0\right)$ = 1.

In this section, we estimate the ability of chains to change their conformations and diffuse, and investigate the mobility of NP in ionic nanocomposites. To calculate the chain’s orientational relaxation time, we measure the autocorrelation function $C_{\mathrm{ee}}\left(t\right)$ [79] of the chain end–end vector $R_{\mathrm{ee}}=r_{N}-r_{1}$ defined by

(6) $$C_{\mathrm{ee}}\left(t\right)=\frac{\langle R_{\mathrm{ee}}\left(t\right)\cdot R_{\mathrm{ee}}\left(0\right)\rangle }{\langle R_{\mathrm{ee}}^{2}\rangle },$$

averaged over all chains and the equilibrium ensemble. The $C_{\mathrm{ee}}\left(t\right)$ results for dispersed systems of N=40 chains are depicted in Figure 6a (end-charged) and Figure 6b (p3-charged chains). These graphs show that the chain relaxation becomes faster as we increase the NP volume fraction, and a comparison between these two graphs suggests that the chain relaxation is slower for p3-charged than end-charged polymers at a given NP volume fraction. In particular, for the p3-charged systems, the chain end–end vector almost never relaxes within a time span of ≈10${}^{5}$ LJ time units at NP volume fractions of ϕ<11.6%. We further calculated $C_{\mathrm{ee}}$ for p3-charged systems with N=100 and N=200, and noticed the exact same behavior for low NP volume fractions (ϕ<11.6%). Therefore in this regime of volume fractions the entire p3-charged nanocomposite system may be identified as a ‘solid’ rather than a ‘melt’. We further complement this statement by additionally investigating the NP and chain center-of-mass mobility, see discussions at the end of this section.

**Figure 7:**
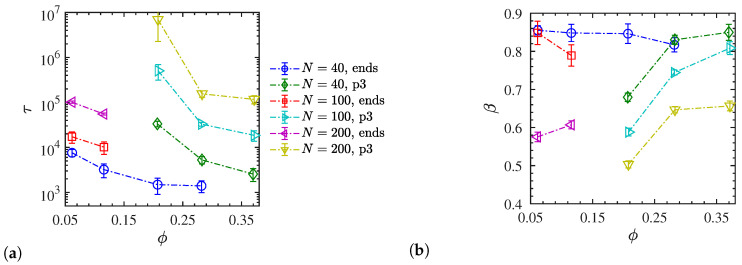
(**a**) Average chain end–end relaxation time τ and (**b**) the corresponding stretched exponent *β* for liquid-like dispersed systems. Dash-dotted lines are guides to the eye.

To quantify the chain’s orientational relaxation for dispersed liquid-like systems, we fit the numerical results of $C_{\mathrm{ee}}\left(t\right)$ to the Kohlrausch–William–Watts stretched exponential function (dash-dotted line in Figure 6b), which is also suitable for describing the relaxation process in soft disordered heterogeneous systems [79,80]. It is given by:

(7) $$C_{\mathrm{ee}}\left(t\right)=\exp\left[-{\left(t/\tau_{r}\right)}^{\beta }\right],$$

where β is the stretched exponent, and $\tau_{r}$ is the orientational relaxation time associated with the stretched exponential function. The average orientational relaxation time τ of the chains is then obtained by integrating Equation (7) as

(8) $$\tau =\int_{0}^{\infty }C_{\mathrm{ee}}\left(t\right)dt=\tau_{r}\left(\frac{1}{\beta }\right)!,$$

where x!=Γ(x+1) denotes the generalized factorial or shifted gamma function. The average relaxation time and the corresponding stretched exponent of all liquid-like dispersed systems are shown in Figure 7a,b, respectively. For a given chain length, the average orientational relaxation time for p3-charged chains exceeds that of end-charged chains, and at a given NP volume fraction, the longer (entangled) chains relax more slowly than the smaller oligomeric ones. The results further indicate that increasing the NP volume fraction makes the chains relax faster, and the change in relaxation time with the NP volume fraction is more drastic for p3-charged chains compared with end-charged chains. Increasing the NP loading makes it more likely for the positively charged chains to find a negatively charged NP in closer proximity, thus enhancing the screening of the electric field, and making the ionic interaction effectively weaker. Consequently, the chains relax faster as reflected by their smaller end–end vector relaxation times. Similarly, changing from p3-charged to end-charged chains at a given NP loading has the same effect. It decreases the polymer charge density and thus makes the electrostatic interactions weaker. The chains can thus move more freely and feature a smaller end-to-end relaxation time τ. The calculated stretched exponents β for the dispersed systems are shown in Figure 7b. Their values are smaller than unity for all systems, meaning that there is a distribution of relaxation times (compared to a mono-exponential decay) associated with the chain relaxation, and β decreases with the increasing chain length. In fact, the trends of the stretched exponent β are exactly the opposite of the mean relaxation time, i.e., the longer the chain relaxation time, the smaller the stretched exponent. Furthermore, while β remains relatively insensitive to the NP loading for end-charged chains, it increases with the NP loading for p3-charged chains, implying faster chain relaxation and reorientation.

**Figure 8:**
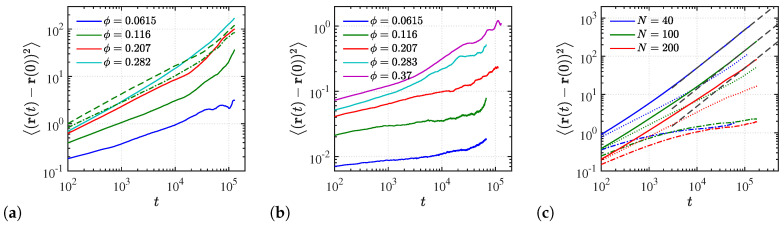
Mean-squared displacement (MSD) of NPs for with (**a**) end-charged and (**b**) p3-charged *N* = 40 chains for dispersed systems at different NP volume fractions. Dashed and dash-dotted lines in (**a**) show the MSD for systems with NP loading of *ϕ* = 11.6% of end-charged chains with polymerization degrees *N* = 200 and *N* = 100, respectively. (**c**) MSD of polymer center-of-mass for NP loading of *ϕ* ≈ 37% (solid lines), *ϕ* = 20.8% (dotted lines), and *ϕ* = 11.6% (dash-dotted lines) for p3-charged chains. Dashed black lines in (**c**) indicate the diffusion limit where MSD(t)∝t.

Next, we focus on translational aspects of NP mobility in these systems. In Figure 8 we show the mean-squared displacements (MSD) of NPs in a polymer matrix composed of N=40 oligomers. Specifically, in Figure 8a,b the dynamics of NPs are compared between matrices of end- and p3-charged polymers. We observe that in both types of matrices the NP MSD increases with increasing NP volume fraction. Such anomalous NP dynamical behavior is opposed to that observed in conventional (non-ionic) nanocomposites containing attractive NPs [81,82], where NP loading hinders the dynamics of the NPs. The anomalous behavior can be explained by the fact that at low NP loading, our NPs contain a higher electrostatic charge in order to retain electrostatic neutrality in the nanocomposite. This leads to a stronger electrostatic attraction between NPs and charged monomers at a smaller ϕ. Moreover, for p3-charged polymers the NP MSD (Figure 8b) is approximately two orders below that for end-charged polymers (Figure 8a). Indeed, in the case of p3-charged polymers, the mobility of NPs becomes negligible, whereas for end-charged systems, at an NP volume fraction ϕ≥11.6%, an NP moves its diameter distance, at least, during the time of measurement. This dynamic discrepancy is driven again by the different electrostatic strengths. In the case of end-charged polymers the total NP electrostatic charge is relatively low. Accordingly, the electrostatic attraction between end-charged polymers and NPs is much lower than between p3-charged polymers and NPs.

Such differences in electrostatic attraction strengths for these two types of charge-sequenced polymers can be quantified by the ratio $\lambda_{B}/R_{\mathrm{charges}}$ between the Bjerrum length ($\lambda_{B}$) and the mean spatial distance between charged monomers ($R_{\mathrm{charges}}$). This dimensionless ratio is known as the Manning number [71], which we adopt here from the field of polyelectrolyte solutions. For p3-charged chains the $R_{\mathrm{charges}}$ is the spatial distance between every three monomers, while for end-charged polymers it coincides with the end-to-end distance $R_{\mathrm{ee}}$.

Furthermore, we observe (Figure 8a) that NP dynamics is fastest in a matrix of end-charged long polymers with N=200 monomers, and slowest in a matrix of end-charged oligomers (N=40). This is in contrast to the dynamical behavior observed in conventional (non-ionic) nanocomposites [82]. In such nanocomposites, NP dynamics is faster in unentangled than in entangled polymer matrices [82]. However, in the present case of ionic nanocomposites, NP dynamics is driven by the electrostatic strength characterized by the dimensionless $\lambda_{B}/R_{\mathrm{charges}}$ ratio. In particular, for end-charged polymers N=200 this number is smaller than for the shorter end-charged polymers N=100, whereas the largest ratio is seen for end-charged oligomers due to their relatively small $R_{\mathrm{ee}}$. The faster dynamics results from the smaller $\lambda_{B}/R_{\mathrm{charges}}$ ratio for the longer chains, while long chains are typically associated with slow dynamics. Another way to alter the $\lambda_{B}/R_{\mathrm{charges}}$ is by altering the relative dielectric constant $\varepsilon_{r}$ while keeping $R_{\mathrm{charges}}$ unchanged. To verify this, we performed additional simulations with a smaller $\varepsilon_{r}=12$ for ϕ=28.2% and observed that NP dynamics is reduced further (results shown in Supplementary Figure S4a) for any polymer length due to the overall increase of dimensionless electrostatic strength ($\lambda_{B}∼\varepsilon_{r}^{-1}$ according to Equation (4)).

Finally, we place our emphasis on the translational aspects of polymer mobility in these systems. The mobilities of the chain’s center-of-mass (COM) for p3-charged chains of different molecular weights are shown in Figure 8c. A comparison between the mobility of chains and mobility of NPs, performed in Figure 8b,c, shows that the chains are more mobile than the NPs. For example, p3-charged chains of N=40 at an NP loading of ϕ=37% exhibit an MSD three orders of magnitude larger than the NP MSD. For the smaller ϕ=11.6% it is one order of magnitude larger in the timespan of ≈10${}^{5}$ time units. Figure 8c further suggests that at the small volume fraction of ϕ=11.6%, the chain COMs do not diffuse and are effectively immobile in the observation timespan (≈10${}^{5}$ time units), as they move less than one atomic diameter, or a fraction of their $R_{g}$. This observation, together with the extremely small NPs mobility, implies a solid-like behavior of the p3-charged systems in the small NP loading regime. In addition, comparing the COM MSDs of chains for different degrees of polymerization at high NP loading, reveals that shorter chains are more mobile in the matrix, and can even reach the diffusion regime (where MSD(t)∝t) within the simulation production time of ≈10${}^{5}$ LJ time units, as indicated by the dashed lines in Figure 8c. Furthermore, the dynamics of chain COMs at ϕ=20.8% is slower than those at ϕ=37% due to the higher electrostatic attraction between ionic polymers and NPs at low NP loading. Such dynamic behavior of ionic nanocomposites is the opposite to the behavior observed in conventional (non-ionic) nanocomposites, where polymer dynamics is hindered by the NP loading [81,82,83]. Furthermore, in nanocomposites with reduced $\varepsilon_{r}=12$ at ϕ=28.2% we observe that the mobility of chain COMs is reduced in comparison to in the case of $\varepsilon_{r}=24$ for any polymer length (results shown in Supplementary Figure S4b) due to the increase in electrostatic strength.

Furthermore, we calculated the MSD of monomers located within the interphase region—which is considered within half $R_{g}$ distance from NP surfaces [84,85]—and compared it with the corresponding values for “bulk” monomers farther away (Supplementary Section 8, Figures S16 and S17). For end-charged chains, the bulk monomer dynamics is rather insensitive to the NP loading, while it increases with NP loading within the interphase. In the case of p3-charged systems, the monomer dynamics enhances within both the interphase and bulk regions with NP loading. The heterogeneity of monomer dynamics between the bulk region and interphase is larger for p3-charged than end-charged chains. In addition, at a given volume fraction ϕ, monomer dynamics within the interphase is found to be slower for unentangled (N=40) than for entangled (N=100, 200) chains.

### 3.4. Polymer–Nanoparticle Temporary Crosslinks

**Figure 9:**
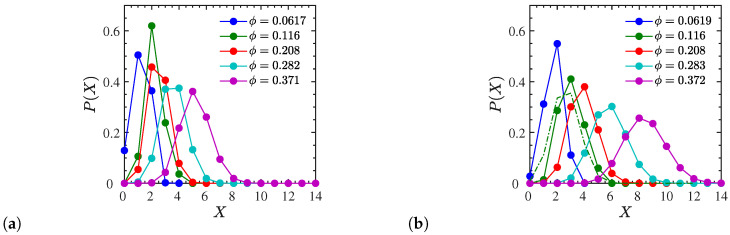
Probability *P*(*X*) of a single p3-charged chain with (**a**) *N* = 100 and (**b**) *N* = 200 monomers to crosslink *X* different NPs simultaneously, normalized such that $\sum_{i}P(X_{i})=1$. Shown are results for dispersed systems at different NP volume fractions. For comparison, the dash-dotted line in (**b**) corresponds to an end-charged system at *ϕ* = 11.6%. The lines are guides to the eye.

In this section, we focus on explaining the different dynamical behaviors observed for the two different types of ionic polymers (end- and p3-charged) studied. To this end we reveal further details of the interaction between the ionic polymers and NPs. In particular, in Figure 9 we show the probability distribution P(X) of a single p3-charged entangled polymer establishing temporary crosslinks [21] with *X* different NPs simultaneously. We define a temporary crosslink between a polymer and an NP to be formed when one (or more) monomer(s) from the chain comes into the r=1 neighborhood of an NP surface. The distribution P(X) becomes broader with NP loading as well as with the chain length *N* (Figure 9). For instance, for the highest NP loading for N=200, there is an extremely high probability of the charged polymers forming temporary crosslinks with eight NPs, while for N=100, they are more likely to form temporary crosslinks with five NPs. By reducing the NP loading, less NPs are involved in the formation of temporary crosslinks, and the distribution becomes more abrupt. P(X) was also calculated for entangled dispersed systems of end-charged polymers. The result for N=200 at ϕ=11.6% is shown in Figure 9b with a dash-dotted line for comparison. The distribution is shifted to the left compared to the p3-charged systems at the same NP loading, suggesting that p3-charged chains are capable of temporarily crosslinking more NPs at a given NP loading. Moreover, for nanocomposites with a lower $\varepsilon_{r}=12$ at ϕ=28.2% we observe that the shape of the distribution P(x) remains unchanged for each polymer length, while the amount of temporary crosslinks is slightly reduced (results shown in Supplementary Figure S5a).

**Figure 10:**
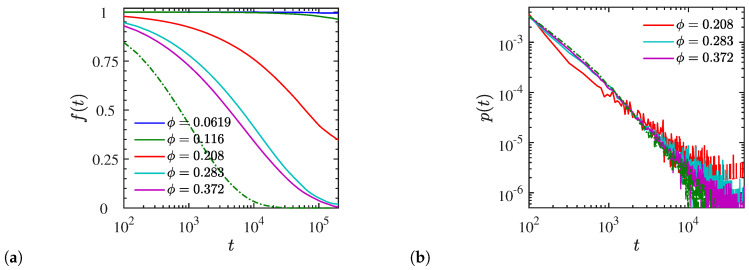
(**a**) Survival probability *f*(*t*) of a polymer–NP crosslink to remain intact at *t* given that it already existed at an arbitrary reference time *t* = 0. (**b**) The corresponding temporary crosslink lifetime distribution *p*(*t*), normalized such that ∫*p*(*t*)*dt* = 1 over the simulation timespan. Data shown for all dispersed systems of *N* = 200 containing p3-charged chains forming either ‘temporary’ or ‘apparent’ crosslinks in (**a**), and only liquid-like systems with temporary crosslinks in (**b**). Dash-dotted lines in (**a**) and (**b**) correspond to a end-charged system at *ϕ* = 11.6%.

In addition, we calculated the probability of a temporary crosslink between polymers and NPs remaining intact (lifetime), f(t) over a certain time *t*, given that the crosslink existed at some reference time t=0 (Figure 10a). A different behavior is observed with the NP loading. At a low NP volume fraction (ϕ≤11.6%), f(t) remains constant over time or p3-polymers, thus implying a formation of ‘apparent’ permanent crosslinks because of the strong electrostatic attraction between charged monomers and highly charged NP surface beads. However, at a high NP volume fraction, f(t) decays by approximately 55% until $t=10^{4}$. This is due to the lower total charge that NPs contain at the high NP loading. As a consequence, the electrostatic attraction between polymers and NPs is weaker at a high NP loading, thus the probability of a polymer–NP temporary crosslink remaining intact reduces with time. Instead, in the case of end-charged polymers f(t) decays much faster although the NP volume fraction is low. This particular result exemplifies the different behavior of the crosslinks formed by differently charged polymers, and explains the different NP dynamics that we observed for the polymer matrices with identical molecular weight (Figure 8). The crosslinks formed between NPs and end-charged polymers cannot be sustained and break down in a shorter time. Moreover, for nanocomposites with a smaller $\varepsilon_{r}=12$ at ϕ=28.2% we observe that f(t) decays more slowly, for instance, it reduces by approximately 20% during $t=10^{4}$ (results available in Supplementary Figure S5b).

We further calculated the lifetime distribution p(t) of temporary crosslinks for liquid-like (ϕ>11.6%) dispersed systems of p3-charged polymers with N=200 (Figure 10b). It indicates that the system with NP loading of ϕ=20.8% exhibits more long-lasting crosslinks with a lifetime t≥2000 than the systems with a higher NP loading. Furthermore, the tails of the distributions ($t>10^{4}$) imply that increasing the NP loading reduces the probability of a prolonged crosslink lifetime. Due to the simultaneous decrease in the electrostatic attraction, this is in accordance with the results we obtained for f(t). The dash-dotted green line in Figure 10b corresponds to the system with an NP loading of ϕ=11.6% in an end-charged N=200 matrix; it indicates a lower probability for long lifetime crosslinks compared with the p3-charged systems. The continuity equation for crosslinks formation/destruction, considering the time reference at t=0 (without the loss of generality) follows from the relation

(9) $$f\left(0\right)-f\left(t\right)=-tf^{\prime }\left(0\right)+\int_{0}^{t}f^{\prime }\left(0\right)\int_{s}^{t}p\left(l-s\right)dl\;ds,$$

where the left-hand side is the normalized number of crosslinks generated and still existing at time *t*. The first term on the right-hand side is the total normalized number of newly generated crosslinks in the timespan of [0,t], and the second term is the fraction of them destroyed when time *t* has passed. After rearranging and differentiating both sides of Equation (9) with respect to *t* using Leibniz’s rule we have

(10) $$p\left(t\right)=-\frac{f^{\prime \prime }\left(t\right)}{f^{\prime }\left(0\right)},$$

which provides an alternative analytical formula to compute the temporary crosslink lifetime distribution p(t) from f(t). We find excellent agreement between this theoretical formula and the numerical results of p(t), see Supplementary Figure S6.

### 3.5. Entanglement Network

**Figure 11:**
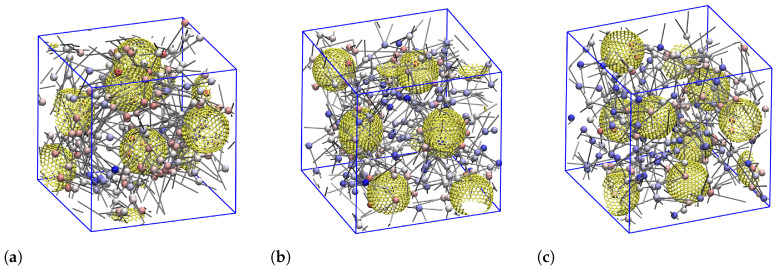
Entanglement network. 3D snapshots of entanglements (colorful spheres) residing on the multiple disconnected shortest path (gray cylinders) for dispersed p3-charged chains of (**a**) *N* = 40, (**b**) *N* = 100, and (**c**) *N* = 200 at an NP volume fraction of *ϕ* ≈ 11.6%. The entanglements (spheres) are colored based on their distance to their closest NP, from red (smallest distance) to blue (largest distance). The polymer chains are not shown here for the sake of clarity. 2D-view snapshots of these three entanglement networks are shown in Supplementary Figure S14.

The observed formation of crosslinks, which result in a temporary network of chains interconnecting different NPs might have an effect on the number of entanglements present in these nanocomposites, as the classical disentanglement process gets distorted. To investigate this issue we performed an entanglement analysis on all dispersed ionic nanocomposites, using the Z1 algorithm [86]. In particular, we calculated the primitive path networks of the charged polymers for all NP loadings, from which we obtain the number of “kinks” considered to be proportional to the number of entanglements per chain. Upon inspecting the positions of the kinks and NPs we can assign each kink to belong to either the group of polymer–polymer entanglements (amount of such kinks per chain denoted as *Z*) or the group of NP–polymer entanglements (denoted as $Z_{\mathrm{NP}}$). Following previous works [87,88,89], we undertook this analysis with two limits: the phantom limit, where NPs are simply ignored, and the frozen limit, where NPs serve as obstacles but do not move during the minimization procedure. Snapshots of ‘frozen’ entanglement networks for selected systems are shown in Figure 11 and Supplementary Figures S14 and S15. They will be discussed further below. Entanglement points residing on the primitive paths are colored by their distance to the closest NP: from red (close to the NP surface) to blue (maximally far away from the NP surface).

**Figure 12:**
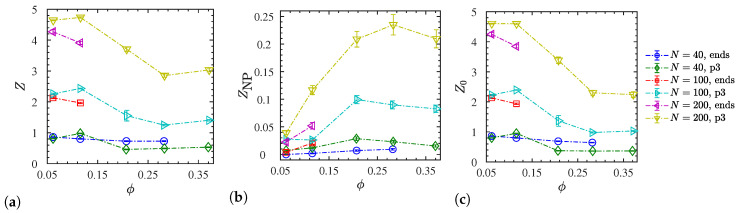
Mean number of (**a**) polymer–polymer entanglements per chain *Z* and (**b**) polymer–NP entanglements per chain *Z*_NP_, both calculated in the frozen NP limit. (**c**) Polymer–polymer entanglements per chain *Z*_0_ in the phantom-limit (NPs ignored in the analysis). Data shown only for dispersed systems as a function of NP volume fraction. Dash-dotted lines are guides to the eye.

The effect of NP volume fraction on the resulting number of entanglements is provided by Figure 12a,b. For the entangled p3-charged systems (N=100, 200), we find that *Z* is almost constant at low NP loadings (ϕ≤11.6%) below the percolation threshold ($\phi_{c}\approx 16$%) [90] and tends to decrease with increased NP loading % (Figure 12c). The p3-charged chains therefore disentangle at ϕ>11.6%. This explains why the chain’s dynamics are getting enhanced (Figure 8c) with increased NP loading up to some NP volume fraction (ϕ=28%). Beyond this fraction, *Z* no longer changes and remaining changes in the translational dynamics of p3-charged polymers must be governed by the presence of NPs, and their interaction with the polymers. In the case of end-charged polymers, we show in Figure 12 that *Z* is also reduced within the available small window, located below the percolation threshold ($\phi_{c}\approx 16$%) [90] ), where the systems are dispersed. In the case of end-charged oligomers, there is only a slight decrease of *Z* with NP loading. In this case, the charge distribution seems to affect the degree of polymer–polymer entanglements for N= 100,200. As can be seen by comparing Figure 12a,c, all these trends are insensitive to the method we use (frozen versus phantom), since there are few polymer–NP entanglements (Figure 12b) [89]. Nevertheless, their amount increases with an NP loading up to ϕ≈28%. Once polymers reside in voids created by neighboring NPs, there will no longer be any NP–polymer entanglements, and $Z_{\mathrm{NP}}$ must ultimately drop to zero. Assuming random walk statistics of the primitive path, the behavior of the degree of polymer entanglements is intimately related to the ratio between the contour length of the primitive path, $L_{\mathrm{pp}}$, and the end-to-end distance of the polymers. The contour length $L_{\mathrm{pp}}^{\mathrm{phantom}}$ of the primitive path in the phantom limit is therefore shown in Supplementary Figure S10 for all charged systems.

**Figure 13:**
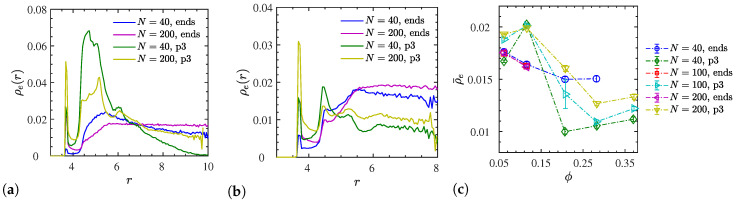
(**a**,**b**) Selected radial number density profiles of entanglements from NP center *ρ*_*e*_(*r*), and (**c**) the average number density of entanglements in the system ${\bar{\rho }}_{e}$ for all dispersed systems (*ε*_*r*_ = 24). Data in (**a**) corresponds to *ϕ* = 11.6% (all dispersed), and in (**b**) to *ϕ* = 28.3% (all dispersed except the end-charged *N* = 200 that had been included for comparison). For the p3-charged systems of (**a**,**b**) snapshots of their entanglement networks are available in Figure 11 and Supplementary Figure S15, respectively.

So far, we have discussed the total number $Z+Z_{\mathrm{NP}}$ of entanglements per chain. The rheologically relevant entanglement number density ${\bar{\rho }}_{e}=\left(Z+Z_{\mathrm{NP}}\right)n/V\left(1-\phi \right)$ is shown in Figure 13c. We also evaluated the underlying radial density $\rho_{e}\left(r\right)$ as a function of the distance to NP centers (Figure 13a,b). This carries information about the density of entanglements that goes far beyond the $Z_{\mathrm{NP}}/Z$ ratio. To make sure that these densities are not biased by the presence of neighboring NPs, we determined $\rho_{e}\left(r\right)$ using entanglements and spherical shell volumes residing within the Voronoi volume of their nearest NP (details in Supplementary Section 7). Density profiles are shown for extreme cases observed at ϕ=11.6% (Figure 13a) and ϕ=28.3% (Figure 13b). At the lower ϕ, the ${\bar{\rho }}_{e}$ reaches its maximum for p3-charged systems, while the situation is reversed at the larger volume fraction. The p3-charged systems show a pronounced tendency for entanglements directly at, or not far from the NP surface. This tendency diminishes with increasing ϕ (note the different scales on the *y*-axes), while the end-charged systems can be considered more homogenous and less sensitive to variations of NP loading. It is only the end-charged N=200 systems that clearly reach a bulk plateau, while the short p3-charged N=40 do not seem to exhibit any entanglements in space half way between NPs (confirmed by Figure 11a). This is supported by their tendency to increase their $R_{g}$ with decreasing ϕ (Figure 5b). The p3-charged oligomers tend to stretch out due to the intramolecular repulsion. The profile suggests that they seem to be almost completely unentangled in the bulk, i.e., far away from the NP surfaces. We confirmed this by independent simulations of a bulk system containing p3-charged chains. The remaining low-ϕ systems shown in Figure 13a have not reached a plateau yet, while their bulk entanglement densities remain finite.

## 4. Conclusions

To summarize, we investigated the structure, conformations and dynamics of unentangled and entangled polymers in ionic nanocomposites, up to approximately 40% NP loading, using a coarse-grained model for ionic polymers and NPs by means of molecular dynamics simulations. We observed structural and dynamical behavior different from that known for conventional (non-ionic) nanocomposites. In particular, we observed that dispersion, NP dynamics, and polymer–NP crosslinks depend on the ratio $\lambda_{B}/R_{\mathrm{charges}}$ rather than on the matrix molecular weight. Specifically, NP dynamics is faster in entangled end-charged polymer matrices than in unentangled end-charged polymer matrices, since the ratio $\lambda_{B}/R_{\mathrm{charges}}$ is smaller. This is the opposite of the dynamic behavior observed in conventional nanocomposites. NP dynamics also depends on the type of matrix that is dispersed, and slows down very dramatically in the case of p3-charged polymers. Moreover, the decrease of the relative dielectric constant of the nanocomposite further influences chain dynamics by altering the electrostatic strength, again characterized by $\lambda_{B}/R_{\mathrm{charges}}$. Temporary crosslinks show a behavior that depends on the type of charged polymer matrix. We have observed that the average radii of gyration of entangled chains (N=200) are unperturbed by charged NPs in ionic nanocomposites at any NP loading up to ϕ=37%, as long as the gyration radius does not exceed the NP diameter, $R_{g}\leq 2r_{\mathrm{NP}}$. The entanglement behavior of long p3-charged chains observed in our study with NP volume fraction is qualitatively similar to conventional (non-ionic nanocomposites) [87,91,92]. The average entanglement number density depends on both the $\lambda_{B}/R_{\mathrm{charges}}$ ratio and matrix molecular weight. We are planning to investigate the amount of entanglements and temporary crosslinks, expected to play important roles in the nonequilibrium mechanical behavior of the ionic nanocomposites, in the near future.

## Figures and Tables

**Table 1:** Lennard–Jones (LJ) units (first four columns), and derived units (remaining columns) used in this manuscript. The constant $\varepsilon_{0}$ denotes the vacuum electric permittivity, $k_{B}$ is Boltzmann’s constant, and *m* the mass of a single monomer bead.

Length	Energy	Mass	Charge	Time	Pressure	Temperature	Spring Coefficient
σ	ϵ	*m*	$\sqrt{4\pi \varepsilon_{0}\sigma \epsilon }$	$\sqrt{m\sigma^{2}/\epsilon }$	$\epsilon /\sigma^{3}$	$\epsilon /k_{B}$	$\epsilon /\sigma^{2}$

**Table 2:** Charged nanocomposite systems studied for matrices containing chains with N=40, N=100, and N=200 beads. NP volume fraction ϕ (%), number $n_{\mathrm{NP}}$ of spherical NPs of identical radius $r_{\mathrm{NP}}$, polymerization degree *N*, number of such chains *n*, and individual NP negative charge *Q*. Charge distribution on chains is denoted by the type attribute. Type ‘ends’ if all polymers chain ends (terminal beads) are charged, and ‘p3’ if every 3rd monomer is periodically charged along the polymer backbone (including chain ends), c.f. Figure 1a. The number of charges per polymer is $n_{\mathrm{NP}}Q/n$ in each case. NP dispersion :: is detected for systems marked by a checkmark. This table is for systems with $\varepsilon_{r}=24$, while systems with $\varepsilon_{r}=12$ had been studied as well. The exact volume fractions are mentioned in the figures, for the purpose of this table they are precise by ±0.1%. Neutral counterparts have been equilibrated in addition for all the listed systems.

ϕ	Charge	Oligomer N=40				Polymer N=100				Polymer N=200			
±0.1%	Type	$n_{\mathrm{NP}}$	*n*	Q/q	::	$n_{\mathrm{NP}}$	*n*	Q/q	::	$n_{\mathrm{NP}}$	*n*	Q/q	::
6.2%	ends	3	270	180	✓	4	144	72	✓	4	72	36	✓
6.2%	p3	3	270	1260	✓	4	144	1224	✓	4	72	1206	✓
11.6%	ends	6	270	90	✓	12	216	36	✓	12	108	18	✓
11.6%	p3	6	270	630	✓	8	144	612	✓	8	72	603	✓
20.8%	ends	12	270	45	✓	24	216	18	×	24	108	9	×
20.8%	p3	12	270	315	✓	16	144	306	✓	16	72	306	✓
28.3%	ends	18	270	30	✓	36	216	12	×	36	108	6	×
28.3%	p3	18	270	210	✓	24	144	204	✓	24	72	201	✓
37.1%	ends	27	270	20	×	36	144	8	×	36	72	4	×
37.1%	p3	27	270	140	✓	36	144	136	✓	36	72	134	✓

## Supplementary Materials

The following are available online at https://www.mdpi.com/2073-4360/12/11/2591/s1, Figure S1: $R_{g}^{2}$ versus *N*, Figure S2: Cumulative radial distribution of NP-NP distances, Figure S3: Additional radial distribution of NP-NP distances, Figure S4: Additional MSDs of NPs and polymers, Figure S5: Additional crosslink and survival probabilities, Figure S6: Test of Equation (10), Figure S7: Analysis of system size effects, Figure S8: Analysis of system size effects, Figure S9: 3D snapshots, Figure S10: Primitive path length $L_{\mathrm{pp}}$, Figure S11: Additional entanglement number density version II profiles, Figure S12: Additional entanglement number density version I and II profiles, Figure S13: Hypothetical number of entanglements around an individual NP, Figure S14: Entanglement networks at ϕ≈11.6%, Figure S15: Entanglement networks at ϕ≈28.3%, Figures S16 and S17: MSD of monomers at interphase and bulk regions.

## References

[1] Crosby A.J., Lee J.Y. (2007). Polymer nanocomposites: The nano effect on mechanical properties. Polym. Rev..

[2] Hu H., Onyebueke L., Abatan A. (2010). Characterizing and modeling mechanical properties of nanocomposites. Review and evaluation. J. Miner. Mater. Charact. Eng..

[3] Suvorova Y.V., Alekseeva S.I., Fronya M.A., Viktorova I.V. (2013). Investigations of physical and mechanical properties of polymeric nanocomposites (Review). Inorg. Mater..

[4] Rong M.Z., Zhang M.Q., Liu H., Zeng H., Wetzel B., Friedrich K. (2001). Microstructure and tribological behavior of polymeric nanocomposites. Indust. Lubric. Tribol..

[5] Clancy T.C., Frankland S.J.V., Hinkley J.A., Gates T.S. (2010). Multiscale modeling of thermal conductivity of polymer/carbon nanocomposites. Int. J. Therm. Sci..

[6] Ganesan V., Jayaraman A. (2014). Theory and simulation studies of effective interactions, phase behavior and morphology in polymer nanocomposites. Soft Matter.

[7] Zheng X., Lin Q., Jiang P., Li Y., Li J. (2018). Ionic liquids incorporating polyamide 6: Miscibility and physical properties. Polymers.

[8] Supova M., Martynkova G.S., Grazina S., Barabaszova K. (2011). Effect of nanofillers dispersion in polymer matrices: A review. Sci. Adv. Mater..

[9] Mackay M.E., Tuteja A., Duxbury P.M., Hawker C.J., Van Horn B., Guan Z., Chen G., Krishnan R.S. (2006). General strategies for nanoparticle dispersion. Science.

[10] Ferdous S.F., Sarker M.F., Adnan A. (2013). Role of nanoparticle dispersion and filler-matrix interface on the matrix dominated failure of rigid C_60_-PE nanocomposites: A molecular dynamics simulation study. Polymer.

[11] Cao X.Z., Merlitz H., Wu C.X., Ungar G., Sommer J.U. (2016). A theoretical study of dispersion-to-aggregation of nanoparticles in adsorbing polymers using molecular dynamics simulations. Nanoscale.

[12] Jouault N., Dalmas F., Boue F., Jestin J. (2012). Multiscale characterization of filler dispersion and origins of mechanical reinforcement in model nanocomposites. Polymer.

[13] Winey K.I., Kashiwagi T., Mu M. (2007). Improving electrical conductivity and thermal properties of polymers by the addition of carbon nanotubes as fillers. MRS Bull..

[14] Karatrantos A., Composto R.J., Winey K.I., Clarke N. (2012). Primitive path network, structure and dynamics of SWCNT/polymer nanocomposites. IOP Conf. Ser. Mat. Sci. Eng..

[15] Samir E., Salah M., Hajjiah A., Shehata N., Fathy M., Hamed A. (2018). Electrospun PVA polymer embedded with ceria nanoparticles as silicon solar cells rear surface coaters for efficiency improvement. Polymers.

[16] Karatrantos A., Clarke N., Kröger M. (2016). Modeling of polymer structure and conformations in polymer nanocomposites from atomistic to mesoscale: A Review. Polym. Rev..

[17] Jankar J., Douglas J.F., Starr F.W., Kumar S.K., Cassagnau P., Lesser A.J., Sternstein S.S., Buehler M.J. (2010). Current issues in research on structure property relationships in polymer nanocomposites. Polymer.

[18] Fernandes N.J., Akbarzadeh J., Peterlik H., Giannelis E.P. (2013). Synthesis and properties of highly dispersed ionic silica-poly(ethylene oxide) nanohybrids. ACS Nano.

[19] Fernandes N.J., Koerner H., Giannelis E.P., Vaia R.A. (2013). Hairy nanoparticle assemblies as one-component functional polymer nanocomposites: Opportunities and challenges. MRS Commun..

[20] Relinque J.J., de Leon A.S., Hernandez-Saz J., Garcia-Romero M.G., Navas-Martos F.J., Morales-Cid G., Molina S.I. (2019). Development of surface-coated polylactic acid/polyhydroxyalkanoate (PLA/PHA) nanocomposites. Polymers.

[21] Odent J., Raquez J.M., Dubois P., Giannelis E.P. (2017). Ultra-stretchable ionic nanocomposites: From dynamic bonding to multi-responsive behaviors. J. Mater. Chem. A.

[22] Odent J., Raquez J.M., Samuel C., Barrau S., Enotiadis A., Dubois P., Giannelis E.P. (2017). Shape-memory behavior of polylactide/silica ionic hybrids. Macromolecules.

[23] Potaufeux J.E., Odent J., Notta-Cuvier D., Delille R., Barrau S., Giannelis E.P., Lauro F., Raquez J.M. (2020). Mechanistic insights on ultra-tough polylactide-based ionic nanocomposites. Compos. Sci. Technol..

[24] Donato K.Z., Matejka L., Mauler R.S., Donato R.K. (2017). Recent applications of ionic liquids in the sol-gel process for polymer-silica nanocomposites with ionic interfaces. Colloids Interfaces.

[25] Yu K.H., Wang D., Wang Q.M. (2018). Tough and self-healable nanocomposite hydrogels for repeatable water treatment. Polymers.

[26] Zou Y.T., Fang L., Chen T.Q., Sun M.L., Lu C.H., Xu Z.Z. (2018). Near-infrared light and solar light activated self-healing epoxy coating having enhanced properties using MXene flakes as multifunctional fillers. Polymers.

[27] Hänninen A., Sarlin E., Lyyra I., Salpavaara T., Kellomäki M., Tuukkanen S. (2018). Nanocellulose and chitosan based films as low cost, green piezoelectric materials. Carbohyd. Polym..

[28] Ruiz de Luzuriaga A., Matxain J.M., Ruipérez F., Martin R., Asua J.M., Cabanero G., Odriozola I. (2016). Transient mechanochromism in epoxy vitrimer composites containing aromatic disulfide crosslinks. J. Mater. Chem. C.

[29] Sessini V., Brox D., Lopez A.J., Urena A., Peroni L. (2018). Thermally activated shape memory behavior of copolymers based on ethylene reinforced with silica nanoparticles. Nanocomposites.

[30] Li T., Li Y., Wang X., Li X., Sun J. (2019). Thermally and near-infrared light-induced shape memory polymers capable of healing mechanical damage and fatigued shape memory function. ACS Appl. Mater. Interfaces.

[31] Wei T., Tang Z., Yu Q., Chen H. (2017). Smart antibacterial surfaces with switchable bacteria-killing and bacteria-releasing capabilities. ACS Appl. Mater. Interfaces.

[32] Zheng Y., Zhang A., Tan Y., Wang N., Yu P. (2013). Property-structure relationship of titania ionic liquid nanofluids. Soft Mater..

[33] Yang S., Tan Y., Yin X., Chen S., Chen D., Wang L., Zhou Y., Xiong C. (2016). Preparation and characterization of monodisperse solvent-free silica nanofluids. J. Dispers. Sci. Technol..

[34] Hong B., Chremos A., Panagiotopoulos A.Z. (2012). Simulations of the structure and dynamics of nanoparticle-based ionic liquids. Faraday Discuss..

[35] Hong B., Panagiotopoulos A.Z. (2013). Diffusivities, viscosities, and conductivities of solvent-free ionically grafted nanoparticles. Soft Matter.

[36] Babayekhorasani F., Dunstan D.E., Krishnamoorti R., Conrad J.C. (2016). Nanoparticle diffusion in crowded and confined media. Soft Matter.

[37] Nath P., Mangal R., Kohle F., Choudhury S., Narayanan S., Wiesner U., Archer L.A. (2018). Dynamics of nanoparticles in entangled polymer solutions. Langmuir.

[38] Poling-Skutvik R., Krishnamoorti R., Conrad J.C. (2015). Size-dependent dynamics of nanoparticles in unentangled polyelectrolyte solutions. ACS Macro Lett..

[39] Karatrantos A., Composto R.J., Winey K.I., Kröger M., Clarke N. (2019). Modeling of entangled polymer diffusion in melts and nanocomposites: A Review. Polymers.

[40] Ma B., Nguyen T.D., Pryamitsyn V.A., de la Cruz M.O. (2018). Ionic correlations in random ionomers. ACS Nano.

[41] Ting C.L., Sorensen-Unruh K.E., Stevens M.J., Frischknecht A.L. (2016). Nonequilibrium simulations of model ionomers in an oscillating electric field. J. Chem. Phys..

[42] Wu S., Xiao C., Zhang Z., Chen Q., Matsumiya Y., Watanabe H. (2018). Molecular design of highly stretchable ionomers. Macromolecules.

[43] Sampath J., Hall L. (2018). Influence of a nanoparticle on the structure and dynamics of model ionomer melts. Soft Matter.

[44] Volgin I.G., Larin S.V., Lyulin A.V., Lyulin S.V. (2018). Coarse-grained molecular-dynamics simulations of nanoparticle diffusion in polymer nanocomposites. Polymer.

[45] David A., Pasquini M., Tartaglino U., Raos G. (2020). A coarse-grained force field for silica–polybutadiene interfaces and nanocomposites. Polymers.

[46] Brini E., Algaer E.A., Ganguly P., Li C., Rodríguez-Ropero F., van der Vegt N. (2013). Systematic coarse-graining methods for soft matter simulations—A review. Soft Matter.

[47] Kremer K., Grest G.S. (1990). Dynamics of entangled linear polymer melts: A molecular-dynamics simulation. J. Chem. Phys..

[48] Kröger M., Hess S. (2000). Rheological evidence for a dynamical crossover in polymer melts via nonequilibrium molecular dynamics. Phys. Rev. Lett..

[49] Rubinstein M., Colby R.H. (2003). Polymer Physics.

[50] Miwatani R., Takahashi K.Z., Arai N. (2020). Performance of coarse graining in estimating polymer properties: Comparison with the atomistic model. Polymers.

[51] Warner H.R. (1972). Kinetic theory and rheology of dilute suspensions of finitely extendible dumbbells. Ind. Eng. Chem. Fund..

[52] Kröger M., Loose W., Hess S. (1993). Structural changes and rheology of polymer melts via nonequilibrium molecular dynamics. J. Rheol..

[53] Hagita K., Murashima T., Iwaoka N. (2018). Thinning approximation for calculating two-dimensional scattering patterns in dissipative particle dynamics simulations under shear flow. Polymers.

[54] Deguchi T., Uehara E. (2017). Statistical and dynamical properties of topological polymers with graphs and ring polymers with knots. Polymers.

[55] Kröger M. (2015). Simple, admissible, and accurate approximants of the inverse Langevin and Brillouin functions, relevant for strong polymer deformations and flows. J. Non-Newtonian Fluid Mech..

[56] Moghimikheirabadi A., Sagis L.M.C., Kröger M., Ilg P. (2019). Gas–liquid phase equilibrium of a model Langmuir monolayer captured by a multiscale approach. Phys. Chem. Chem. Phys..

[57] Allen M.P., Tildesley D.J. (1987). Computer Simulation of Liquids.

[58] Hou J.X., Svaneborg C., Everaers R., Grest G.S. (2010). Stress relaxation in entangled polymer melts. Phys. Rev. Lett..

[59] Hoy R.S., Foteinopoulou K., Kröger M. (2009). Topological analysis of polymeric melts: Chain-length effects and fast-converging estimators for entanglement length. Phys. Rev. E.

[60] Norris A.N., Sheng P., Callegari A.J. (1985). Effective medium theories for two-phase dielectric medium. J. Appl. Phys..

[61] Cheng Y., Chen X., Wu K., Wu S., Chen Y., Meng Y. (2008). Modeling and simulation for effective permittivity of two-phase disordered composites. J. Appl. Phys..

[62] Plimpton S. (1995). Fast parallel algorithms for short-range molecular dynamics. J. Comp. Phys..

[63] Hagita K., Morita H., Doi M., Takano H. (2016). Coarse-grained molecular dynamics simulation of filled polymer nanocomposites under uniaxial elongation. Macromolecules.

[64] Hagita K., Morita H., Takano H. (2016). Molecular dynamics simulation study of a fracture of filler-filled polymer nanocomposites. Polymer.

[65] Humphrey W., Dalke A., Schulten K. (1996). VMD–Visual Molecular Dynamics. J. Mol. Graph..

[66] Karatrantos A., Clarke N., Composto R.J., Winey K.I. (2015). Polymer conformations in polymer nanocomposites containing spherical nanoparticles. Soft Matter.

[67] Moghimikheirabadi A., Sagis L.M., Ilg P. (2018). Effective interaction potentials for model amphiphilic surfactants adsorbed at fluid–fluid interfaces. Phys. Chem. Chem. Phys..

[68] Zhang Q., Archer L.A. (2002). Poly(ethylene oxide)/silica nanocomposites: Structure and rheology. Langmuir.

[69] Koizumi N., Hanai T. (1964). Dielectric properties of polyethylene glycols: Dielectric relaxation in solid state. Bull. Inst. Chem. Res..

[70] Porter C., Boyd R. (1971). A dielectric study of the effects of melting on molecular relaxation in poly(ethylene oxide) and polyoxymethylene. Macromolecules.

[71] Manning G.S. (1969). Limiting laws and counterion condensation in polyelectrolyte solutions I. Colligative properties. J. Chem. Phys..

[72] Crawford M.K., Smalley R.J., Cohen G., Hogan B., Wood B., Kumar S.K., Melnichenko Y.B., He L., Guise W., Hammouda B. (2013). Chain conformation in polymer nanocomposites with uniformly dispersed nanoparticles. Phys. Rev. Lett..

[73] Tuteja A., Duxbury P.M., Mackay M.E. (2008). Polymer chain swelling induced by dispersed nanoparticles. Phys. Rev. Lett..

[74] Karatrantos A., Koutsawa Y., Dubois P., Clarke N., Kröger M. (2018). Miscibility and diffusion in ionic nanocomposites. Polymers.

[75] Sen S., Xie Y., Kumar S.K., Yang H., Bansal A., Ho D.L., Hall L., Hooper J.B., Schweizer K.S. (2007). Chain conformations and bound-layer correlations in polymer nanocomposites. Phys. Rev. Lett..

[76] Jouault N., Dalmas F., Said S., Schweins R., Jestin J., Boue F. (2010). Direct measurement of polymer chain conformation in well-controlled model nanocomposites by combining SANS and SAXS. Macromolecules.

[77] Jouault N., Crawford M.K., Chi C., Smalley R.J., Wood B., Jestin J., Melnichenko Y.B., He L., Guise W.E., Kumar S.K. (2016). Polymer chain behavior in polymer nanocomposites with attractive interactions. ACS Macro Lett..

[78] Jouault N., Kumar S.K., Smalley R.J., Chi C., Moneta R., Wood B., Salerno H., Melnichenko Y.B., He L., Guise W.E. (2018). Do very small POSS nanoparticles perturb s–PMMA chain conformations?. Macromolecules.

[79] Moghimikheirabadi A., Ilg P., Sagis L.M.C., Kröger M. (2020). Surface rheology and structure of model triblock copolymers at a liquid–vapor interface: A molecular dynamics study. Macromolecules.

[80] Sagis L.M.C., Liu B., Li Y., Essers J., Yang J., Moghimikheirabadi A., Hinderink E., Berton-Carabin C., Schroen K. (2019). Dynamic heterogeneity in complex interfaces of soft interface-dominated materials. Sci. Rep..

[81] Karatrantos A., Clarke N., Composto R.J., Winey K.I. (2014). Structure, entanglements and dynamics of polymer nanocomposites containing spherical nanoparticles. IOP Conf. Ser. Mat. Sci. Eng..

[82] Karatrantos A., Composto R.J., Winey K.I., Clarke N. (2017). Polymer and spherical nanoparticle diffusion in nanocomposites. J. Chem. Phys..

[83] Lin C.C., Gam S., Meth J.S., Clarke N., Winey K.I., Composto R.J. (2013). Do attractive polymer-nanoparticle interactions retard polymer diffusion in nanocomposites. Macromolecules.

[84] Holt A.P., Griffin P.J., Bocharova V., Agapov A.L., Imel A.E., Dadmun M.D., Sangoro J.R., Sokolov A.P. (2014). Dynamics at the polymer/nanoparticle interface in poly(2-vinylpyridine)/silica nanocomposites. Macromolecules.

[85] Jouault N., Moll J.F., Meng D., Windsor K., Ramcharan S., Kearney C., Kumar S.K. (2013). Bound polymer layers in nanocomposites. ACS Macro Lett..

[86] Kröger M. (2005). Shortest multiple disconnected path for the analysis of entanglements in two- and three-dimensional polymeric systems. Comput. Phys. Commun..

[87] Li Y., Kröger M., Liu W.K. (2012). Nanoparticle effect on the dynamics of polymer chains and their entanglement network. Phys. Rev. Lett..

[88] Toepperwein G.N., Karayiannis N.C., Riggleman R.A., Kröger M., de Pablo J.J. (2011). Influence of nanorod inclusions on structure and primitive path network of polymer nanocomposites at equilibrium and under deformation. Macromolecules.

[89] Karatrantos A., Clarke N., Composto R.J., Winey K.I. (2016). Entanglements in polymer nanocomposites containing spherical nanoparticles. Soft Matter.

[90] Thorpe M.F., Phillips J.C. (2001). Phase Transitions and Self-Organization in Electronic and Molecular Networks.

[91] Toepperwein G.N., Riggleman R.A., de Pablo J.J. (2011). Dynamics and deformation response of rod-containing nanocomposites. Macromolecules.

[92] Schneider G.J., Nusser K., Willner L., Falus P., Richter D. (2011). Dynamics of entangled chains in polymer nanocomposites. Macromolecules.

