# Supplementary material for: Polymer Conformations, Entanglements and Dynamics in Ionic Nanocomposites: A Molecular Dynamics Study
Source: Polymers (Basel). 2020 Nov 4;12(11):2591. doi: 10.3390/polym12112591 (PMC7694256; doi:10.3390/polym12112591)
Supplement: Supplementary file 1 [file polymers-12-02591-s001.pdf]

Supplementary Materials: Polymer conformations, entanglements and dynamics in ionic nanocomposites: A molecular dynamics study

Ahmad Moghimikheirabadi [1*], Clément Mugemana [2], Martin Kröger [1*] and Argyrios V. Karatrantos [2*]

Contents

1 Single chain statistics 1

2 NP coordination number 2

3 Effect of electrostatic strength 2

4 Crosslink life time distribution 4

5 System-size analysis 4

6 Snapshots of the super structure formation 4

7 Primitive path network 6

8 Monomer MSD within interphase and bulk 10

All the results reported here correspond to the systems with an effective dielectric constant of $\varepsilon_r = 24$, unless otherwise stated.

1. Single chain statistics

Squared mean radius of gyration $\langle R_g \rangle^2$ and squared mean end-end distance $\langle R_{\mathrm{ee}} \rangle^2$ as a function of polymerization degree are indicated in Figs. S1a and S1b respectively. These systems behave like ideal chains where $\langle R_g \rangle^2$, $\langle R_{\mathrm{ee}} \rangle^2 \propto N$, and the chains remain basically ideal with the charges and with NP loading. There seems to be an exception for $N = 40$ charged systems, where the charges play a role for the local conformation at different NP loadings. While chains are locally more stiff for longer p3-charged chains, this does not translate into a larger R_g for large N.

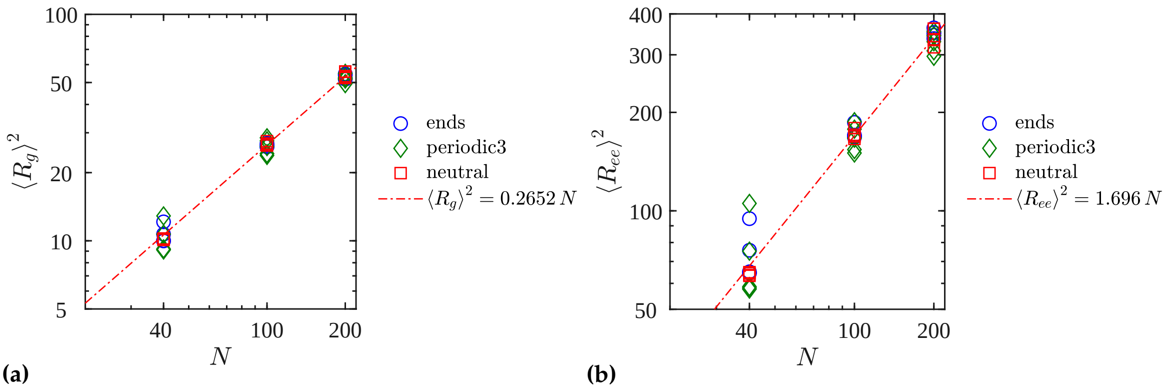

Figure S1. Squared mean radius of gyration $\langle R_g \rangle^2$ **(a)** and Squared mean end-end distance $\langle R_{\text{ee}} \rangle^2$ **(b)** as a function of polymerization degree for neutral (squares), end-charged (circles), and p3-charged (diamonds) chains. For each chain type and N, the data for five different NP loading ranging from $\phi = 6.2 - 37.2\%$ is plotted in each graphs. Dash-dotted line in each graph shows the linear scaling for neutral chains in nanocomposites where $\langle R_g \rangle^2$, $\langle R_{\text{ee}} \rangle^2 \propto N$.

2. NP coordination number

The average number of neighboring NPs located around a given NP up to a radial distance r (from its center) can be calculated through integrating the NP center–NP center radial distribution function as

$$\#\text{NP}(r) = 4\pi\phi / V_{\text{NP}} \int_0^r r'^2 g(r') dr', \tag{1}$$

and the results for p3-charged dispersed systems of $N = 100$ and $N = 200$ are shown in Figs. S2a and S2b respectively.

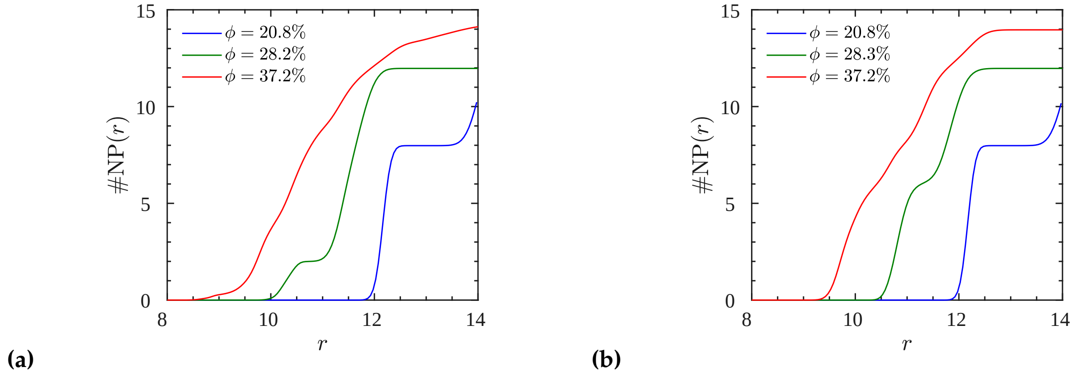

Figure S2. Average number of NPs, #NP(r), located up to radial distance r from a given NP center in the system. Dispersed p3-charged systems of **(a)** $N = 100$ and **(b)** $N = 200$.

3. Effect of electrostatic strength

We further studied the effect of electrostatic strength via varying the dielectric constant from $\varepsilon_r = 24$ down to 12, thus increasing the Bjerrum length from $\lambda_\text{B} = 2.1$ to 4.2, resulting in a stronger electrostatic interaction (Figures S3–S5).

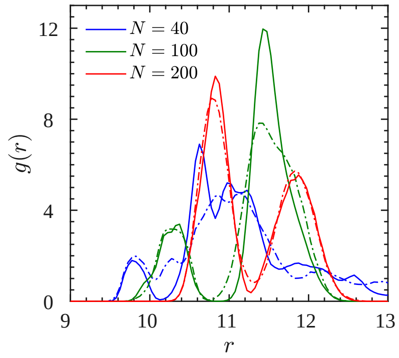

Figure S3. NP center–NP center radial distribution function for dispersed p3-charged systems at NP loading of $\phi = 28.2$ %. Solid lines correspond to the systems with dielectric constant of $\varepsilon_r = 12$, and dash-dotted lines to the systems with $\varepsilon_r = 24$.

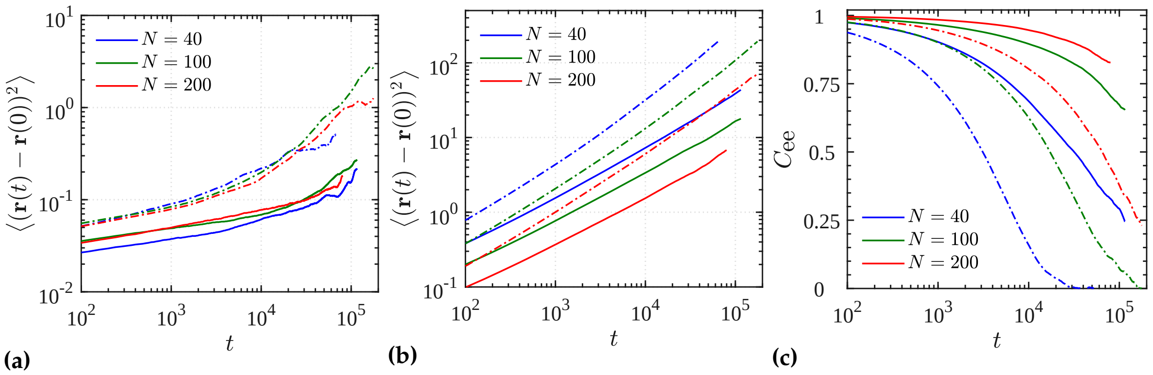

Figure S4. (a) MSD of NPs, **(b)** MSD of polymer COMs, and **(c)** chain end-end vector auto-correlation function for dispersed p3-charged systems at NP loading of $\phi = 28.2$ %. Solid lines correspond to the systems with dielectric constant of $\varepsilon_r = 12$, and dash-dotted lines to the systems with $\varepsilon_r = 24$.

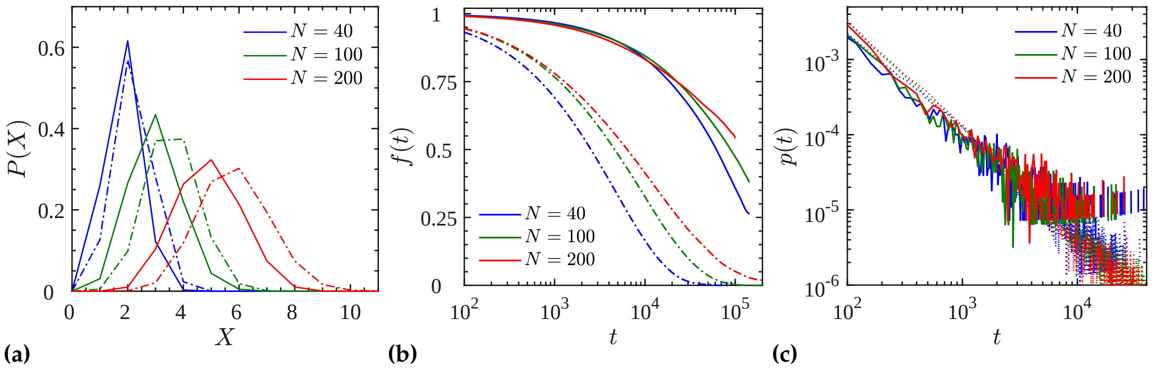

Figure S5. (a) Probability $P(X)$ of a chain to crosslink X different NPs, **(b)** survival probability of a crosslink over time $f(t)$, and **(c)** crosslink life time distribution $p(t)$ for dispersed p3-charged systems at NP loading of $\phi = 28.2$ %. Solid lines correspond to the systems with dielectric constant of $\varepsilon_r = 12$. Dash-dotted lines in (a) and (b), and dotted lines in (c) correspond to the systems with $\varepsilon_r = 24$.

4. Crosslink life time distribution

Here we verify the analytical relation $p(t) = -f''(t)/f'(0)$ by comparing $p(t)$ directly calculated from simulation, and its equivalent calculated by numerical derivations of $f(t)$, see Figure S6.

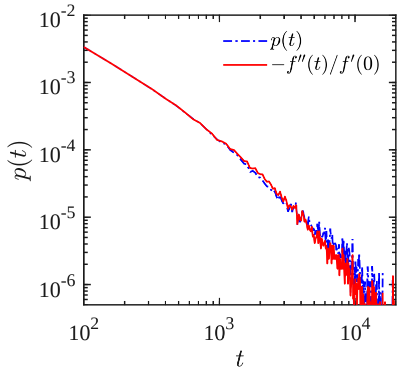

Figure S6. A comparison between crosslink life time distribution $p(t)$ obtained directly from simulation (dash-dotted line), and from analytical formula $-f''(t)/f'(0)$ (solid line) for a p3-charged dispersed system of $N = 200$ at NP loading of $\phi = 11.6$ %.

5. System-size analysis

In order to study the effect of system size on simulation results, we simulated two systems at the same volume fraction of $\phi = 28.3\%$ but with different sizes. A small system consisting of $n = 72$ end-charged chains of $N = 200$ and $n_{\mathrm{NP}} = 24$ NPs, and a larger one with $n = 108$ and $n_{\mathrm{NP}} = 36$. The former system with box dimensions of $29.04 \times 29.04 \times 29.04$, and the latter with $33.26 \times 33.26 \times 33.26$, that is $\approx 50\%$ larger in volume. We found an excellent agreement in both static and dynamic properties between these two systems as described in Figures S7 and S8 respectively. This indicates that the systems considered throughout this study are indeed large enough that the finite-size effects are negligible.

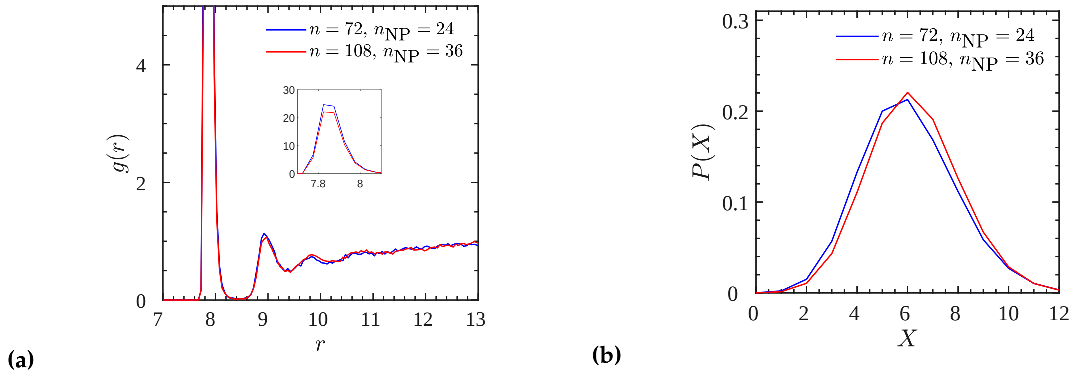

Figure S7. System-size dependence study of static properties such as NP center–NP center radial distribution function **(a)**, and crosslinking probability $P(X)$ **(b)**. Both systems contain end-charged $N = 200$ chains, at the same volume fraction of 28.3%. The red lines correspond to a system that is $\approx 50\%$ larger in volume than the one shown by blue lines.

6. Snapshots of the super structure formation

Here we present a new set of snapshots of the $N = 100$ p3-charged chains in Figure S9, corresponding to the exact coordinates as was shown in the manuscript Figure 4.

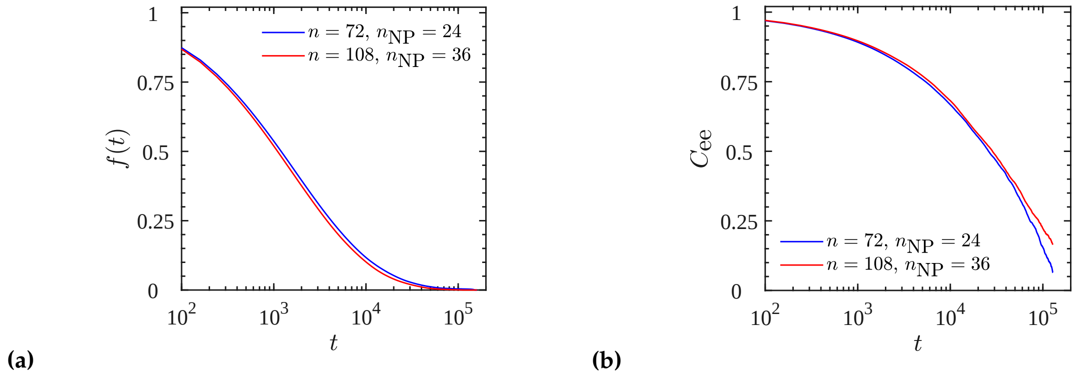

Figure S8. System-size dependence study of dynamic properties such as crosslinking survival probability $f(t)$ **(a)**, and chain end-end vector autocorrelation function **(b)**. Both systems contain end-charged $N = 200$ chains, at the same volume fraction of 28.3%. The red lines correspond to a system that is $\approx$ 50% larger in volume than the one shown by blue lines.

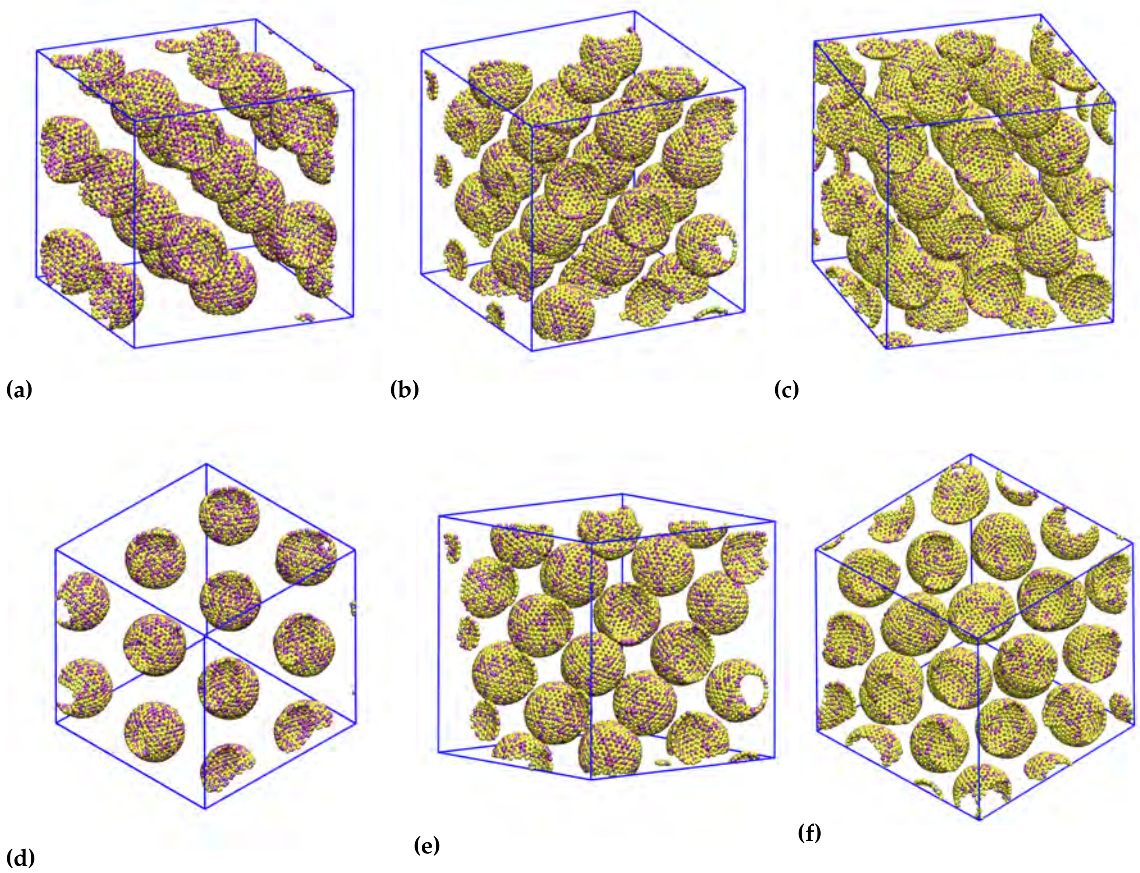

Figure S9. 3D orthographic view snapshots of $N = 100$ p3-charged systems at the NP volume fractions of **(a)** $\phi = 20.8\%$, **(b)** $\phi = 28.2\%$, and **(c)** $\phi = 37.2\%$. 2D projections of these systems were shown in Figures 4c–4e of the manuscript. **(d,e,f)** The bottom row shows the same systems from another viewing angles. Only NPs are shown here for the sake of clarity. Yellow and purple spheres stand for the neutral and charged surface beads respectively.

7. Primitive path network

Here we show additional information about the primitive paths calculated via the Z1 code. The mean contour length of the primitive paths is shown in Figure S10, and the snapshots of entanglement networks are shown in Figures S14 and S15.

We can think of two different versions to calculate a radial density profile of entanglements in the nanocomposites at a certain resolution of Δr (we have chosen $\Delta r = 0.05$). We followed both routes, results in the manuscript are presented for version II. While version I is analogous to the calculation of a pair correlation function taking into account all distances in the system, version II focuses on a density profile that might be more close to the density profile one would obtain for a single NP in a melt, and less affected by ϕ than version I.

Version I: Record (i) the histogram of distances between NPs and entanglements and (ii) the histogram of available volume for monomers as function of distance from NP centers. To calculate an available volume at distance r, the one not occupied by NPs, we perform a Monte Carlo simulation, where we shoot randomly into a shell of thickness Δr a distance r away from an NP center. The entanglement density at r is then the mean number of entanglements at r divided by the mean available volume at r, where the maximum r equals half the simulation box size.

Version II: For all entanglements, record their distances r to their closest NP, resulting in a histogram $h_e(r)$ of shell occupancies. To be able to calculate a r-dependent density using this information collected for all entanglements, we need the corresponding r-shell volume around all NPs, that is potentially available for such entanglements. The available volume around an individual NP equals the part of the volume of the shell that overlaps with the voronoi volume of the NP. Phrased differently, the available volume equals the part of the volume of the shell that is closer to the NP than to any other NP. To this end we shoot randomly into the box volume, and accumulate a histogram $h_s(r)$ of distances to the nearest NP. The entanglement density at r is then $h_e(r)/h_s(r)$ times the number density of shots.

Both versions give different results for r beyond the minimum distance between NPs. Consider for example two NPs at distance d, and all entanglements located on their surfaces. Version I leads to a density profile with spikes at $r = r_{\rm NP}$ and $r = r_{\rm NP} + d$, while version II does not exhibit the 2nd peak at the larger r. Note that while the mean number density of entanglements is given by $\bar{\rho}_e = (Z + Z_{\rm NP})n/V(1-\phi)$, it cannot be recovered by simply integrating over $\rho_e(r)$, see Figure S13, as $\rho_e(r)$ as opposed to $h_e(r)$ does not carry information about the amount of entanglements residing at a certain r (Figure S12). Few of the density profiles (predominantly at low ϕ) obtained with version II for oligomers tend to vanish at finite r. We have performed additional, independent simulations of bulk systems containing end-charged and p3-charged systems, to rationalize this observation. Indeed, the p3-charged oligomeric chains almost completely disentangle in the bulk, due to the intramolecular repulsion. This is what version II implies, while version I (Figure S11) yields a plateau at large r due to the presence of the other NPs with their clouds of entanglements.

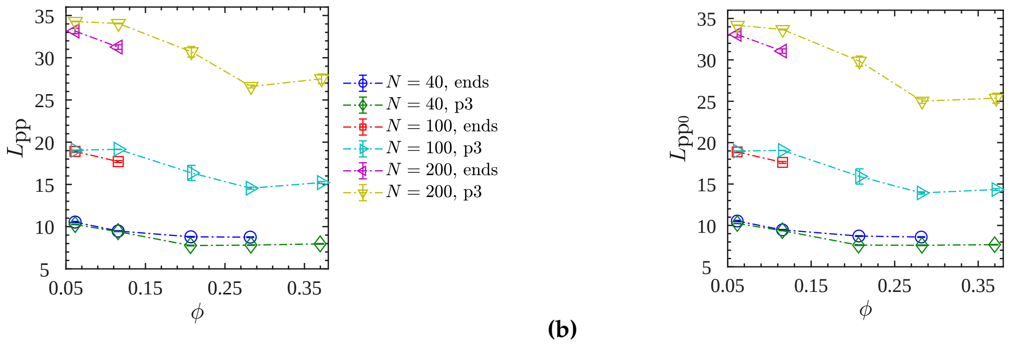

(a) **(b)**

Figure S10. Primitive path length per chain L_{pp} **(a)** in the frozen limit and **(b)** in the phantom-limit (NPs ignored) for all dispersed systems as a function of NP volume fraction ϕ. Dash-dotted lines are guides to the eye.

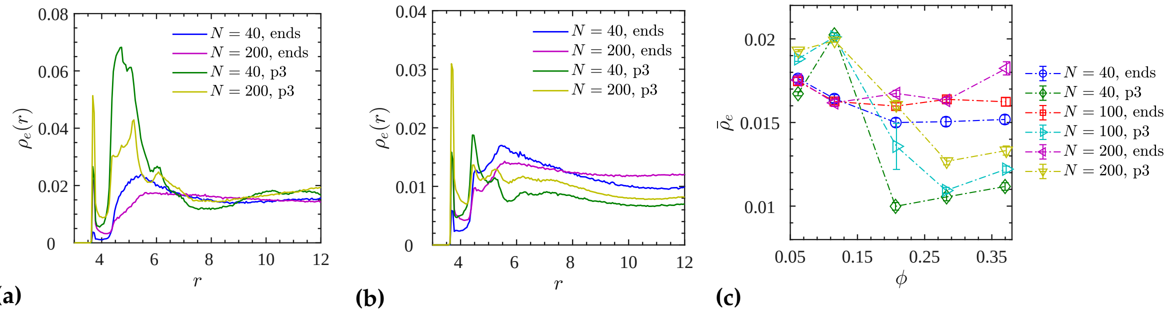

(a) **(b)** **(c)**

Figure S11. **(a,b)** Selected radial number density profiles of entanglements from NP center $\rho_e(r)$ obtained from Version I algorithm, and **(c)** the average number density of entanglements in the system $\bar{\rho}_e$ for all (dispersed and not dispersed) systems ($\varepsilon_r = 24$). Data in (a) corresponds to $\phi = 11.6\%$ (all dispersed), and in (b) to $\phi = 28.3\%$ (all dispersed except the end-charged $N = 200$ that had been included for comparison).

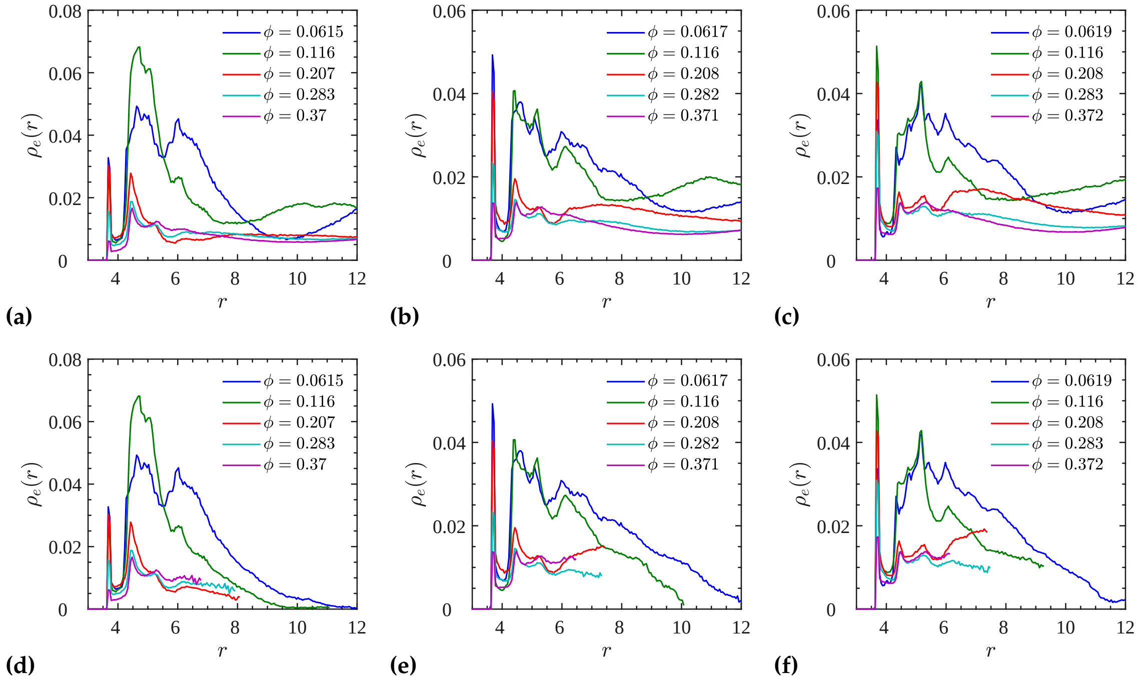

Figure S12. Radial number density profiles of entanglements from NP center $\rho_e(r)$ obtained from Version I (top row) and Version II (bottom row) algorithms for **(a, d)** $N = 40$, **(b, e)** $N = 100$, and **(c, f)** $N = 200$ p3 charged-chains. All cases shown here are dispersed. Version II curves terminate by construction at a distance r that corresponds to the maximum half distance between NPs, while version I curves are not evaluated beyond half box size. Up to minimum half distance between NPs, both versions coincide.

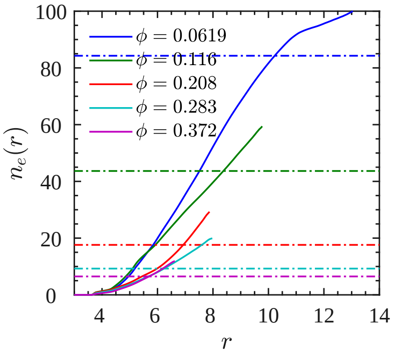

Figure S13. Hypothetical number of entanglements up to distance r from a single NP center $n_e(r) = 4\pi \int_0^r \rho_e(r') r'^2 dr'$ obtained from integrating $\rho_e(r)$ results of V2 algorithm for p3-charged $N = 200$ polymers, as shown in Figure S12f. Dash-dotted lines show the corresponding values of $(Z + Z_{\rm NP})n/n_{\rm NP}$. Every solid lines crosses its dashed lines at a value for r that corresponds to the mean linear size of an NP Voronoi volume (in the absence of polymers), and terminates at the maximum half distance between NPs. The true number of entanglements up to a certain r is contained in the histogram $h_e(r)$.

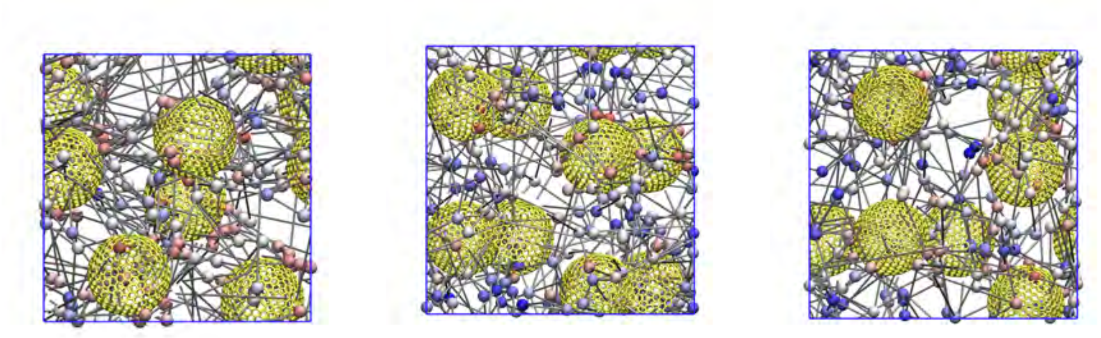

(a) **(b)** **(c)**

Figure S14. 2D-view snapshots of entanglements (colorful spheres) and multiple disconnected shortest path (gray cylinders) for dispersed p3-charged chains of **(a)** $N = 40$, **(b)** $N = 100$, and **(c)** $N = 200$ at NP volume fraction of $\phi \approx 11.6\%$. 3D snapshots of the same systems with the exact coordinates are shown in the main text (Figure 11). Entanglement points (spheres) are colored based on their distance to their closest NP, from red (smallest distance) to blue (largest distance). The polymer chains are not shown here for the sake of clarity.

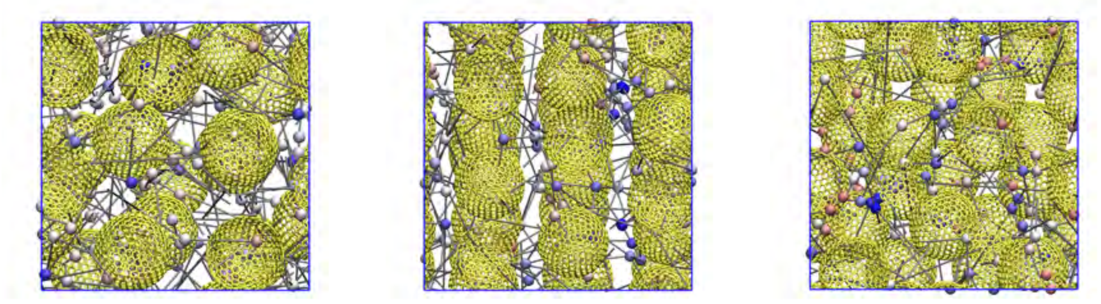

(a) **(b)** **(c)**

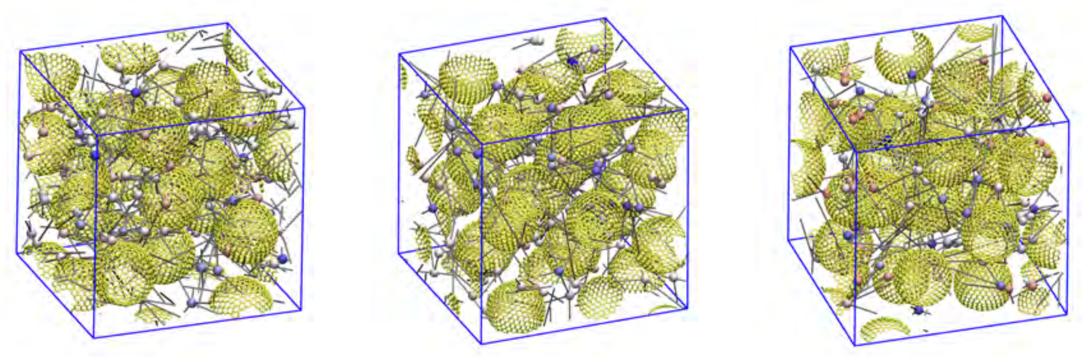

(d) **(e)** **(f)**

Figure S15. Additional snapshots of the entanglement network for dispersed p3-charged chains of **(a,d)** $N = 40$, **(b,e)** $N = 100$, and **(c,f)** $N = 200$ at an NP volume fraction of $\phi \approx 28.3\%$.

8. Monomer MSD within interphase and bulk

In a postprocessing step using the time-averaged gyration radii for all systems, we investigated the polymer dynamics close to the NP surface (interphase region within $R_g/2$ neighborhood of NP surfaces) and in "bulk" (farther than $R_g/2$ from NP surfaces), through calculation of the MSD of monomers in these regions. A comparison between MSD of monomers within the interphase (solid lines) and bulk (dash-dotted lines) for dispersed systems of $N = 40$ oligomers is shown in Figures S16a and S16b for end- and p3-charged chains, respectively. There is a heterogeneity of monomer dynamics within bulk and interphase, which is more drastic for p3-charged than end-charged chains. For end-charged chains (Figure S16a) the monomer mobility in the bulk is rather insensitive to the NP loading, while it enhances within the interphase by increasing the NP volume fraction. This mobility enhancement close to the NP-polymer interface is more drastic for p3-charged chains as indicated in Figure S16b. The reason for this behavior is that at higher volume fractions, the NPs carry less charge on their surfaces (due to the overall system charge neutrality), and hence are less sticky to the oppositely charged monomers at their vicinity.

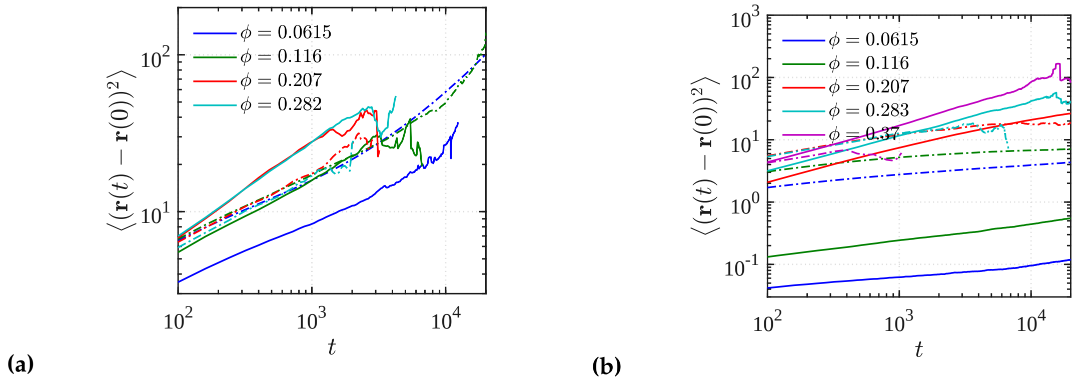

Figure S16. MSD of monomers at interphase (solid lines) and bulk regions (dash-dotted lines) for dispersed systems of **(a)** end-charged and **(b)** p3-charged chains of $N = 40$.

A comparison between the monomer MSD of entangled and unentangled networks within interphase and bulk at an NP volume fraction of $\phi \approx 6.2\%$ is shown in Figures S17a and S17b for end- and p3-charged chains, respectively. It indicates that the monomer mobility within the interphase is slower for unentangled ($N = 40$) than for entangled ($N = 100, 200$) networks for both p3- and end-charged chains. Likewise, monomer bulk mobility seems to depend on the entanglement state as well, especially for p3-charged chains; the entangled networks show a stronger bulk mobility.

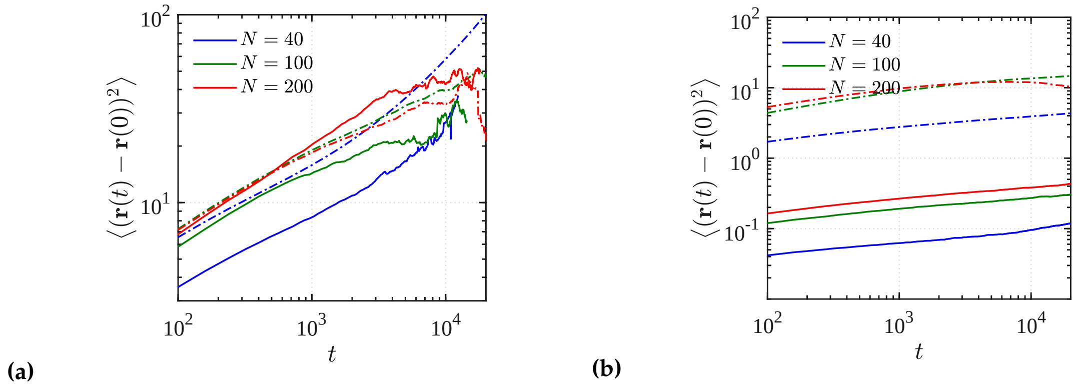

Figure S17. MSD of monomers at interphase (solid lines) and bulk regions (dash-dotted lines) for dispersed systems of **(a)** end-charged and **(b)** p3-charged chains at an NP volume fraction of $\phi \approx 6.2\%$.
